# Meningeal lymphatic vessel dysfunction exacerbates brain injury in CVST mice via endoplasmic reticulum and oxidative stress pathways

**DOI:** 10.3389/fimmu.2026.1745066

**Published:** 2026-01-30

**Authors:** Jianbin Ying, Jun Li, Xianqun Wu, Xuanjie Chen, Hao Zhang, Liangfeng Wei, Junjie Jing, Shousen Wang

**Affiliations:** 1 Department of Neurosurgery, Fuzong Clinical Medical College of Fujian Medical University (900TH Hospital), Fuzhou, China; 2 Department of Neurosurgery, Fujian Children’s Hospital (Fujian Branch of Shanghai Children’s Medical Center), College of Clinical Medicine for Obstetrics & Gynecology and Pediatrics, Fujian Medical University, Fuzhou, China

**Keywords:** brain injury, cerebral venous sinus thrombosis, endoplasmic reticulum stress, meningeal lymphatic vessels, neuroinflammation, oxidative stress

## Abstract

**Objective:**

Meningeal lymphatic vessels (mLVs) play a significant role in neurological homeostasis and disease. However, their contribution to brain injury following cerebral venous sinus thrombosis (CVST) remains unknown. This study investigated whether mLV dysfunction influences the pathological progression of CVST by regulating the endoplasmic reticulum (ER) and oxidative stress(OS)pathways.

**Method:**

A total of 65 male C57BL/6J mice were randomly assigned to four groups: sham-operated, CVST; CVST combined with cervical lymph node ligation (CVST + Ligation); and 4-phenylbutyric acid (4-PBA) intervention. The CVST model was established by inducing thrombosis in the superior sagittal sinus. All sample collection and experimental assays were performed at 2 days post-modeling. Neurobehavioral assessment, histopathological staining, immunofluorescence, western blotting, reverse transcription quantitative polymerase chain reaction, enzyme-linked immunosorbent assay, and bioinformatics analyses were employed to comprehensively evaluate neurological function, brain injury, inflammatory response, key molecular expression in ER/oxidative stress pathways, and alterations in related signaling pathways following mLV dysfunction.

**Result:**

Compared to the CVST group, mice in the CVST+Ligation group exhibited more severe neurological deficits, aggravated histopathological brain injury, increased neuronal loss, and enhanced cellular apoptosis. Transcriptomic analysis following lymphatic dysfunction revealed significant enrichment of pathways related to inflammatory response, cytokine-cytokine receptor interaction, and endoplasmic reticulum (ER) stress. At the levels of immunofluorescence, ELISA, Western blot, and mRNA expression, lymphatic ligation significantly upregulated markers of ER stress and microglial activation/apoptosis (including GRP78, CHOP, ATF4, p-eIF2α, NLRP3, and IL-1β) (*P* < 0.05), as well as downstream apoptosis-related proteins (such as PUMA and Caspase-12) (*P* < 0.05). It also promoted the release of pro-inflammatory cytokines (IL-6, IL-1β, TNF-α, and IL-17) (*P* < 0.05). Administration of the ER stress inhibitor 4-PBA effectively reversed these molecular alterations and significantly alleviated brain injury and neuroinflammation in CVST+Ligation mice.

**Conclusion:**

Dysfunction of mLVs exacerbates brain injury after CVST by promoting neuroinflammation via the ER and oxidative stress pathways. Therapeutically targeting mLVs may represent promising strategies for managing CVST-related neurological injury.

## 1 Introduction

Cerebral venous sinus thrombosis (CVST) is a rare form of cerebrovascular disease, accounting for approximately 0.5% to 1% of all strokes. It is more prevalent in children and adolescents (1–3). Its etiology is complex, with known risk factors including the puerperium, use of oral contraceptives, and systemic inflammation (2–4). In recent years, the incidence of CVST has shown a gradual increase, partly associated with outbreaks of certain respiratory infections (5). CVST has a non-specific clinical presentation that depends primarily on the extent and location of venous obstruction, which often results in non-specific symptoms and a high rate of misdiagnosis. Current therapeutic options remain limited, with anticoagulant therapy being the mainstay of treatment (6). Prognosis varies considerably among patients, and timely restoration of venous outflow is essential to minimize irreversible brain injury and long-term neurological deficits.

In 2015, Louveau et al. (7) identified and validated the existence of meningeal lymphatic vessels (mLVs) in mice and clarified their roles in the development and progression of neurological disorders. In addition to facilitating cerebrospinal fluid drainage and the clearance of metabolic waste, mLVs provide a direct communication pathway between the central and peripheral immune systems, thereby contributing to immune surveillance and maintenance of intracranial immune homeostasis (8–10). Extensive literature indicates that mLVs play a vital role in controlling neuroinflammation following traumatic brain injury and other neurological disorders. In mouse models of traumatic brain injury, enhancing meningeal lymphatic function reduces inflammation and mitigates cognitive impairment (11). Similarly, in models of arterial stroke, meningeal lymphatics influence the post-stroke inflammatory response and, consequently, functional recovery (12). In subarachnoid hemorrhage, impaired meningeal lymphatic function exacerbates cerebral edema, inflammation, and neuronal apoptosis, thereby worsening brain injury (13).

The mechanisms underlying CVST-induced brain injury involve multiple interrelated pathophysiological processes that form a vicious cycle. Thrombosis within the cerebral venous system leads to stenosis or occlusion of the sinus cavities, impairing venous cerebral circulation and obstructing cerebrospinal fluid absorption. The resulting venous hypertension compromises the blood–brain barrier and induces ischemic-hypoxic injury to the brain tissue (14). Damage to the parenchyma and blood-brain barrier further triggers the activation of inflammatory cells such as microglia and neutrophils, which contribute to the inflammatory cascade. Activated microglia and neutrophils release multiple cytokines, including interleukin(IL)-1, IL-6, and tumor necrosis factor (TNF) (15). The secretion of these cytokines exacerbates neuronal injury. Furthermore, inflammasomes such as NLRP3 become activated, promoting the maturation and release of inflammatory mediators and inducing neuronal pyroptosis (16). Using a CVST mouse model, Ding et al. (16) found that endoplasmic reticulum (ER) and oxidative stress can jointly activate inflammasome pathways and trigger pyroptosis following CVST. During ER stress, certain signaling molecules directly activate inflammatory pathways. For example, misfolded or unfolded proteins produced under these conditions activate the NLRP3 inflammasome, a multiprotein complex that promotes the maturation and secretion of the inflammatory cytokines IL-1β and IL-18, thereby inducing local or systemic inflammatory responses (17–19). However, the potential role of mLVs in cerebral venous disorders such as CVST—and the mechanisms underlying their effects—remain largely unexplored. This study aimed to investigate whether mLVs influence the inflammatory process following CVST (post 2 days) via activation of ER and oxidative stress, thereby affecting prognosis. This study further sought to identify novel therapeutic approaches for mitigating CVST-induced brain injury.

## 2 Materials and methods

### 2.1 Animals

A total of 65 male C57BL/6J mice (8–10 weeks old, weighing 22–25 g) were supplied by the Animal Experiment Center of the 900th Hospital. All animals were housed under specific pathogen-free conditions with a fixed 12-h light/dark cycle, ambient temperature maintained at 22 ± 1 C, and relative humidity between 45% and 55%. Standard rodent chow and water were provided *ad libitum* throughout the experimental period. All animal procedures were approved by the Ethics Committee of the 900th Hospital and conducted in accordance with the National Institutes of Health Guide for the Care and Use of Laboratory Animals (Approval No. 2020-051).

### 2.2 Experimental design

65 mice were randomly divided into four group: Control (n=14), CVST (n=17), CVST+ligation (n=17), and 4-PBA (n=17). All sample collection and experimental assays were performed at 2 days post-modeling. In each group, 14 mice were used for histopathological examinations, including H&E staining, Nissl staining, molecular pathology analysis, and immunofluorescence assays. Behavioral testing was performed before tissue collection. The remaining 3 mice per group(CVST,CVST+Ligation, CVST+Ligation+4PBA)were allocated for transcriptome sequencing. Mice that did not meet experimental criteria were excluded and replaced to maintain group sizes. Exclusion criteria were: accidental cerebral contusion/laceration during bone window creation over the superior sagittal sinus; excessive hemorrhage during model establishment; subarachnoid hemorrhage; marked postoperative weight loss; or death from other unexplained causes (**Supplementary Figure 1**).

In this study, anesthesia was induced and maintained using inhaled isoflurane (3% for induction, 1.5% for maintenance), with the depth of anesthesia continuously monitored throughout the procedure. The analgesic regimen consisted of preoperative subcutaneous injection of butorphanol (1 mg/kg) administered 30 minutes prior to surgery as preemptive analgesia. This was followed by repeated doses every 12 hours for a total duration of 24 hours postoperatively. During the perioperative period, core body temperature (maintained at 36.5 ± 0.5°C using a heating pad), heart rate (normal range: 400–550 beats per minute), and respiratory rate (normal range: 80–120 breaths per minute) were continuously monitored using a physiological monitoring system. Respiratory rhythm and depth were also closely observed. During the recovery phase, mice were placed individually in pre-warmed recovery cages with continuous thermal support. Their state of consciousness, locomotor activity, and signs of pain were assessed hourly until full recovery was achieved.

### 2.3 Induction of superior sagittal sinus thrombosis

The superior sagittal sinus (SSS) thrombosis model was established as previously described with minor modifications (20, 21).After positioning in a stereotaxic frame with body temperature maintenance, a 15 mm midline scalp incision was made to expose the skull. The galea and periosteum were dissected to visualize cranial landmarks. A 0.7 mm-wide bone window was created over the SSS using a high-speed drill under stereomicroscopic guidance, with continuous saline irrigation to prevent thermal injury. The thinned bone was removed to expose the SSS. A 6 mm segment of 5–0 suture thread (Aesicang, SA82G, 0.149 mm), pre-soaked in 40% ferric chloride, was placed on the exposed sinus for 2 minutes. After removal, the area was rinsed with saline. Successful CVST induction was confirmed by absence of sinus blood flow after 10 minutes of observation. The incision was sutured, and mice were kept warm until recovery before individual housing. Sham-operated mice underwent SSS exposure without thrombus induction.

### 2.4 Ligation of the deep cervical lymph nodes

Mice were secured in a supine position on a foam board with limbs and jaw immobilized to fully expose the neck. After skin preparation and disinfection with iodine tincture, a 1.5–2 cm midline incision was made from the upper sternum to the mandible. The bilateral superficial cervical lymph nodes were identified along the midline in a figure-of-eight arrangement. Deep cervical lymph nodes were located around the carotid sheath, adjacent to the trachea and thyroid gland. Using microsurgical instruments under microscopic guidance, lymphatic vessels and vascular structures at the lymph node hilum were carefully exposed. The lymph node was elevated, and a 10–0 microsuture was passed around the hilar tissue and securely ligated. Special care was taken to preserve the carotid arteries and recurrent laryngeal nerve. Post-ligation vascular integrity was verified using laser speckle imaging. The incision was subsequently closed, and mice were maintained on a heating pad until full recovery before returning to their home cages.

### 2.5 4-PBA

4-Phenylbutyric acid (4-PBA; Sigma-Aldrich, USA) was administered as an endoplasmic reticulum stress inhibitor via intraperitoneal injection at a dose of 100 mg/kg in physiological saline, 30 minutes prior to modelling. Control mice received an equal volume of solvent. As an endoplasmic reticulum (ER) stress inhibitor, its primary mechanism of action is not to selectively inhibit any specific signaling pathway (such as IRE1, PERK, or ATF6), but to comprehensively alleviate ER stress as a whole, thereby indirectly suppressing the activation of all three pathways (22).

### 2.6 Laser speckle imaging

Cerebral blood flow was assessed using laser speckle contrast imaging two days post-modeling. Mice were positioned with the skull fixed horizontally, and the cranial surface was kept moist with periodic saline application to maintain optical clarity. Any soft tissue proliferation over the superior sagittal sinus was carefully removed with microforceps prior to imaging. Regions of interest (ROIs) were defined as one area within the infarct zone on each side of the midline and two areas along adjacent pontine veins. Relative cerebral blood flow was calculated as the ratio of post-modeling flow values to preoperative baseline measurements. All images were acquired under consistent distance and field conditions after confirming hemostasis, and data were stored for subsequent statistical analysis.

### 2.7 Neurobehavioral assessment: open field test

Prior to testing, all mice were acclimated to the experimental room for at least 2 hours under dim ambient lighting, with the open field arena overhead light maintained continuously illuminated. At the start of each trial, mice were gently placed in the top right corner of the arena. Behavior was recorded for 5 minutes using an automated video tracking system to quantify total distance moved, time spent in the center versus periphery, and grooming episodes. After each session, mice were removed and transferred to clean cages. The arena was thoroughly cleaned with medical-grade alcohol and dried with paper towels to eliminate olfactory cues between trials.

### 2.8 Preparation of paraffin sections

Following collection, mouse brain tissue specimens were fixed in 4% paraformaldehyde at room temperature for 24 h. After routine dehydration and embedding, coronal sections of 5 μm thickness were prepared. Sections were dewaxed and subsequently stained with hematoxylin and eosin (H&E), Nissl (Solae Bio, Cat. No. G1434), or TUNEL (Biyuntian, Cat. No. C1088) according to standard protocols. H&E and Nissl-stained sections were imaged using an optical microscope (M8, PreciPoint GmbH, Germany), while TUNEL-stained sections were visualized under a fluorescence microscope (Lionheart, BioTek, VT, USA).

### 2.9 Immunofluorescence staining

Paraffin sections were prepared as described above. After deparaffinization and rehydration, antigen retrieval was performed using citrate-EDTA buffer (Zhongshan Jinqiao, Cat. No. ZLI-9067) under high-pressure heating for 2 minutes. Sections were then blocked with 3% BSA for 30 minutes at room temperature, followed by stepwise incubation with primary antibodies (GFAP 1:300, Ab4674, Abcam; Neun 1:1000, Ab177487, Abcam; Iba-1 1:500, 019-19741, Wako; iNOS 1:200, 22226-1-AP, Proteintech; CD206 1:200, 24595S, CST) and corresponding TSA fluorophores according to the multiplex staining kit protocol. After stripping, nuclei were counterstained with DAPI, and sections were mounted with anti-fade mounting medium.

Paraffin sections were prepared as described above. After deparaffinization and rehydration, antigen retrieval was performed using citrate-EDTA buffer (Zhongshan Jinqiao, Cat. No. ZLI-9067) under high-pressure heating for 2 minutes. Sections were then blocked with 5% goat serum (Zsbio, Cat. No. ZLI-9022, China) for 30 minutes at room temperature, followed by incubation with the following primary antibodies at 37°C for 1 hour: rabbit anti-CHOP (1:200, 15204-1-AP, Proteintech); rabbit anti-ATF4 (1:200, ET1612-7, Huabio); rabbit anti-PUMA (1:100, 98672T, CST); rabbit anti-Caspase-12 (1:200, A22864, Clona); rabbit anti-CD68(1:200 97778S, CST);anti- Bcl-2(1:400 68103-1-lg, Proteintech); rabbit anti-Bax(1:500 ab32503, abcam);rabbit anti-NLRP3(1:100 ab270449 abcam);anti- IL-1β(1:100 HA601002 Huabio). After PBS washes, corresponding fluorescence-conjugated secondary antibodies (FITC-conjugated goat anti-rabbit IgG (H+L), 1:500, A0562) were added and incubated at 37°C for 1 hour. Following PBS washes, nuclei were counterstained with DAPI (C1006, Beyotime, China) for 10 minutes. All sections were scanned and imaged using a Panoramic SCAN 3D digital pathology slide scanner (3DHISTECH, Hungary) with fluorescence channels. Each experimental group comprised n = 5 independent biological replicates. For each animal, n = 5 fields of view were randomly selected. Data from technical replicates (fields of view) were averaged to represent a single value per biological sample. Statistical significance was determined using an unpaired *t*-test in GraphPad Prism 8.

### 2.10 Immunofluorescence staining of meningeal tissue

Mice were transcardially perfused with ice-cold PBS followed by 4% paraformaldehyde. To visualize the complete meningeal lymphatic network, the skin and neck muscles were carefully dissected from the skull. After removing the cranial portions of the mandible and maxilla, the cranial vault was excised using surgical scissors and fixed intact with the attached dura mater in 4% paraformaldehyde at 4°C for 24 h. The dura mater was then meticulously dissected from the cranial vault using micro-scissors, forceps, and a 27-gauge needle.

Whole-mount meningeal preparations were blocked in solution containing 3% BSA, 0.5% Triton X-100, and 2% fetal bovine serum for 1 h at room temperature, then incubated with primary antibodies (anti-LYVE-1, 1:50, Proteintech 28321-1-AP; anti-fibrinogen, 1:50, Proteintech 15841-1-AP) in blocking buffer at 4°C overnight. After three 8-min PBS washes, tissues were incubated with Alexa Fluor 594-conjugated goat anti-rabbit IgG (1:200, Proteintech RGAR004) for 1 h at room temperature, followed by DAPI (1:1000) counterstaining for 10 min. Following final washes, specimens were mounted on glass slides and imaged using confocal microscopy. Quantitative analysis was performed with ImageJ software.

### 2.11 RNA sequencing and transcriptome analysis

Part of the RNA sequencing data utilized in this study were derived from previously published work (23) (PMID: 37918797), generated from the same CVST (C57BL/6J-Tyrc-Hsd) mouse model. The corresponding raw data are publicly available in the NCBI Gene Expression Omnibus (GEO) under accession number GSE245455.

The newly measured transcriptome data (analyzed by Fujian Airda Biotechnology Co., Ltd.) was obtained by extracting total RNA from mice in three groups (CVST, CVST+Ligation, and 4-PBA group) using TRIzol reagent (Invitrogen, USA) on day 2 post-modeling. RNA quality and concentration were determined using an Agilent 5300 Bioanalyzer and a Thermo Fisher ND-2000 spectrophotometer. cDNA libraries were prepared and sequenced on the NovaSeq X Plus platform (PE150) with NovaSeg Reagent Kits (Majorbio, China). Raw reads were quality-controlled with fastp, aligned to the reference genome using HISAT2 (orientation mode), and assembled with StringTie. Transcript abundance was quantified as TPM (transcripts per million) using RSEM. Transcriptome analysis was completed for 9 samples, yielding a total of 26.93 GB of Clean Data, with each sample achieving a Clean Data volume of 2.99GB and a Q30 base percentage of 97.02% or higher. Sequence alignment was performed between the Clean Reads of each sample and the designated reference genome, with alignment efficiency ranging from 99.14% to 99.22%. Based on the alignment results, gene expression analysis was conducted. Differential expression genes were identified according to gene expression levels across different samples, followed by functional annotation and enrichment analysis.

### 2.12 Western blot

Frozen brain tissues (50 mg) were homogenized in 500 μL of ice-cold RIPA lysis buffer containing protease and phosphatase inhibitors (P1005, Beyotime) using an electric homogenizer. The homogenates were incubated on a shaking platform at 4°C for 30 min and centrifuged at 12,000 ×g for 15 min at 4°C. Supernatants were collected, and protein concentrations were determined using the BCA method (Enhanced BCA Protein Assay Kit, P0009, Beyotime). Protein samples (5 μg/μL) were separated by SDS-PAGE and transferred to PVDF membranes.

After blocking with 5% BSA for 2 h at room temperature, membranes were incubated overnight at 4°C with the following primary antibodies: GRP78/BIP (1:3000, 66574-1-Ig, Proteintech), CHOP (1:1000, 66741-1-Ig, Proteintech), caspase 12 (1:1000, 55238-1-AP, Proteintech), ATF4 (1:1000, 10835-1-AP, Proteintech), phospho-eIF2α (Ser51) (1:1000, #3398, CST) PUMA (1:1000, 55120-1-AP, Proteintech), Phospho-PERK/EIF2AK3 (Thr982) (1:5000, 82534-1-RR, Proteintech), GAPDH (1:20000, 60004-1-Ig, Proteintech) and beta actin (1:10000, 66009-1-Ig, Proteintech). Following TBST washes, membranes were incubated with IRDye 800CW-conjugated secondary antibodies (1:50000, 926-32211/926-32210, LI-COR) for 2 h at room temperature. Protein bands were visualized using an Odyssey Li-COR CLx imaging system. All Western blot quantifications were performed using ImageJ. For each blot, background subtraction was applied to the raw band intensities, followed by normalization to the corresponding loading control in each lane.

### 2.13 RT-qPCR

Take 10 mg of mouse brain tissue and use TRIzol™ (15596026CN, Thermo Scientific, MA, USA) to extract total RNA according to the manufacturer’s instructions. Dilute the RNA to 1 μg/μl with ddH_2_O. Using the reverse transcription kit (HisyGo All-in-One RT Red SuperMix for qPCR (+gDNA Wiper, RT333-01, Vazyme, Nanjing, China), generate cDNA from 1 μg of total RNA according to the manufacturer’s instructions. Use 100 ng of the cDNA as a template for the qPCR reaction. Use ChamQ Universal SYBR qPCR Master Mix (Q711-02, Vazyme, Nanjing, China) as the enzyme for the reaction. The qPCR program comprises 40 cycles of 95°C pre-denaturation for 30 seconds, 95°C denaturation for 10 seconds and 60°C extension for 30 seconds. Reactions were performed on an Applied Biosystems QuantStudio 6 Flex qPCR system. Primers were designed using Primer Premier 5 software (Premier Biosoft International, Palo Alto, CA, USA) and the sequences are provided in **Supplementary Table 1**.

### 2.14 ELISA

Serum concentrations of IL-6, IL-1β, TNF-α, IL-17, and IL-10 were quantified using commercial ELISA kits (J24111, J24057, J24082, J24056, J24126; JILID, Wuhan, China) according to the manufacturer’s protocols. All assays were performed in duplicate, and absorbance values were measured to determine cytokine concentrations.

### 2.15 Statistics analysis

Data are presented as mean ± standard error of the mean (SEM). Statistical analyses were conducted using SPSS 20.0 (IBM, USA). Intergroup comparisons were performed using one-way ANOVA followed by LSD or Dunnett’s T3 *post hoc* tests for multiple comparisons. Independent two-group comparisons were analyzed by Student’s t-test. Correlation analyses utilized Pearson’s correlation coefficient (with data log-transformed when normality assumptions were not met). A threshold of *P* < 0.05 was considered statistically significant. All analyses were performed with investigators blinded to group assignments.

## 3 Result

### 3.1 Dysfunction of mLVs exacerbates brain injury in CVST mice

**Figure 1:**
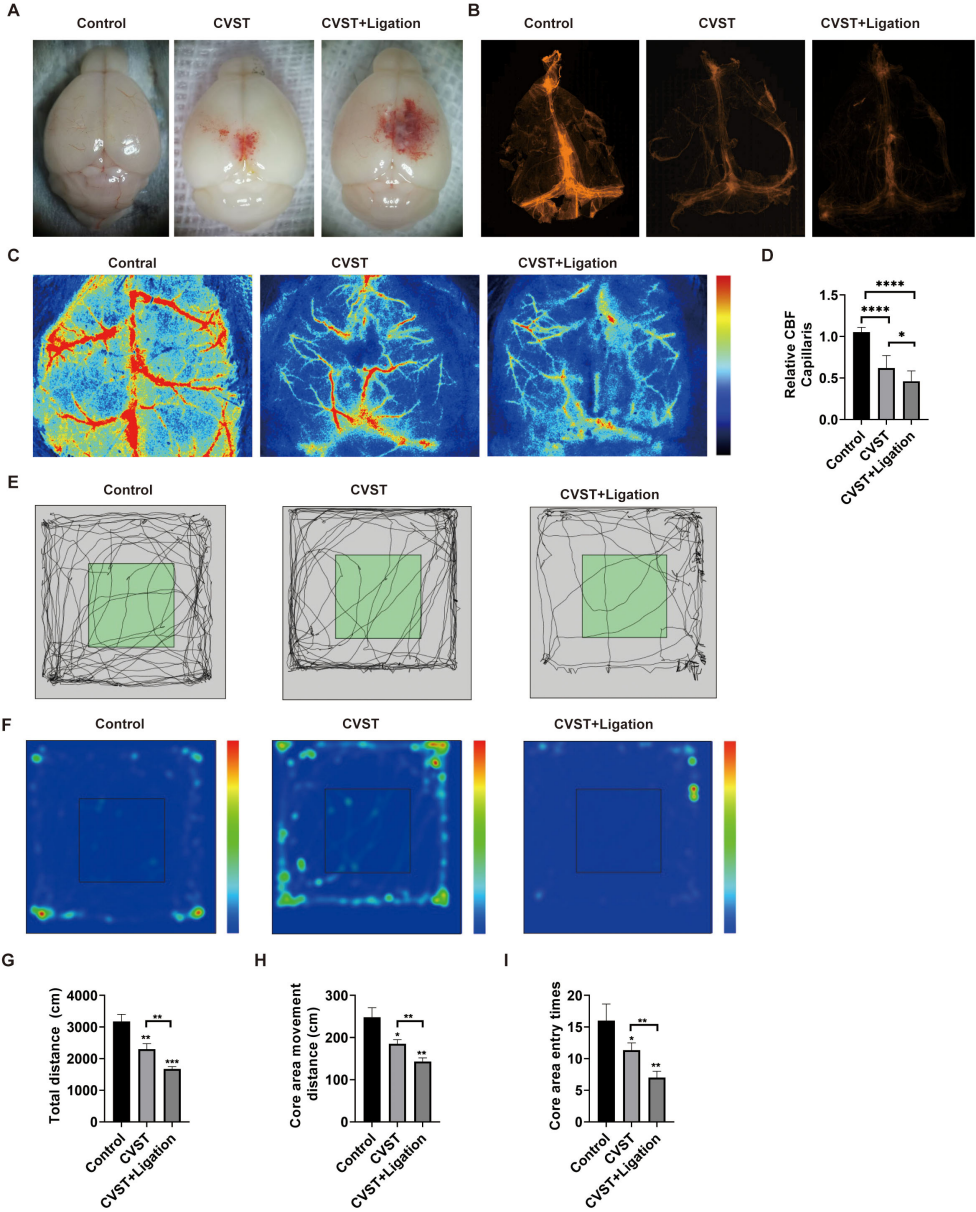
mLVs dysfunction further exacerbates cerebral injury and lymphatic vessel damage in CVST. **(A)** Representative images of dorsal cortical injury in mice at 2 days post-CVST induction, demonstrating that lymphatic dysfunction exacerbates cortical damage. **(B)** Immunofluorescence staining of meningeal lymphatic vessels at 2 days post-CVST induction, revealing that CVST impairs meningeal lymphatic function, which is further diminished following lymphatic ligation. **(C, D)** Laser speckle contrast imaging and quantification of relative cerebral blood flow in peri-infarct ROIs at 2 days post-induction, indicating that CVST reduces cortical perfusion, an effect worsened by lymphatic dysfunction. **(E-I)** Open field test performance at 2 days post-CVST induction. Representative track plots **(E)** and activity heatmaps **(F)** illustrate locomotor patterns in sham, CVST, and CVST with lymphatic ligation groups. Quantitative analysis shows group comparisons of total locomotor distance **(G)**, distance traveled in the central zone **(H)**, and time spent in the central zone **(I)**. These data demonstrate that meningeal lymphatic dysfunction exacerbates neurological deficits in CVST mice. ns, *P* > 0.05; *P < 0.05, **P < 0.01,***P < 0.001, ****P < 0.0001.

Both CVST and CVST+Ligation groups exacerbated brain injury, with the CVST+Ligation group exhibiting more severe damage (**Figure 1A**). Lymphatic ligation also significantly impaired meningeal lymphatic function (**Figure 1B**). Furthermore, laser speckle imaging revealed significantly reduced cerebral blood flow perfusion in the CVST+Ligation group compared to the CVST group (**Figures 1C, D**). The open field test further demonstrated that lymphatic ligation markedly exacerbated neurofunctional deficits in CVST mice (**Figures 1E-I**).

**Figure 2:**
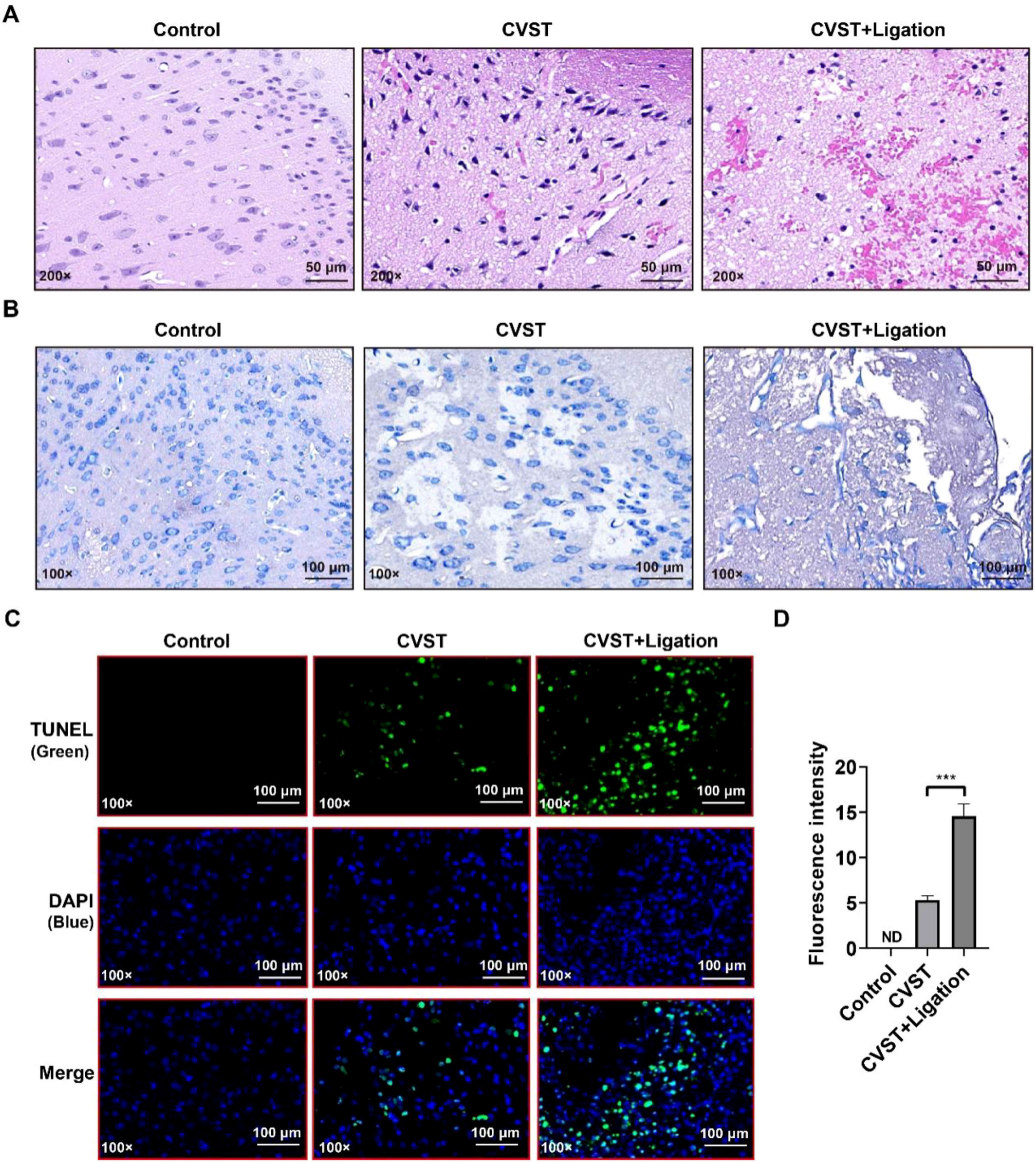
mLVs dysfunction exacerbates CVST-induced cerebral injury and apoptosis. **(A)** Representative HE staining images of brain tissue damage in CVST and CVST+Ligation mice. CVST mice exhibited focal hemorrhage and mild structural disorganization, while the CVST+Ligation group displayed more severe pathological injury, indicating that lymphatic dysfunction exacerbates ischemic and hemorrhagic brain damage. The scale bars represent 50 μm. **(B)** Nissl staining showing neuronal injury in CVST and CVST+Ligation mice. Nissl bodies were markedly reduced in the CVST group, with a more severe reduction observed in the CVST+Ligation group, suggesting that lymphatic dysfunction aggravates neuronal damage. The scale bars represent 100 μm. **(C)** TUNEL staining for apoptosis detection in brain tissue (n=5). TUNEL-positive nuclei (green) were significantly increased in the CVST group and further elevated in the CVST+Ligation group, demonstrating that lymphatic dysfunction induces more severe apoptosis. **(D)** Quantification of TUNEL fluorescence intensity from **(C)** using ImageJ. Statistical analysis was performed using unpaired t-test in GraphPad Prism 8. ***P < 0.001. ND: Not detected. The scale bars represent 100 μm.

Histopathological analysis of mouse brain tissue revealed that HE and Nissl staining both showed brain injury with hemorrhage, neuronal shrinkage, and decreased neuronal density in the CVST and CVST+Ligation groups, with ligation further exacerbating CVST-induced cerebral hemorrhage and neuronal damage (**Figures 2A, B**). Additionally, TUNEL assay demonstrated that both groups induced apoptosis in brain tissue cells, with the ligation group exhibiting more severe apoptotic cell death (**Figures 2C, D**).

**Figure 3:**
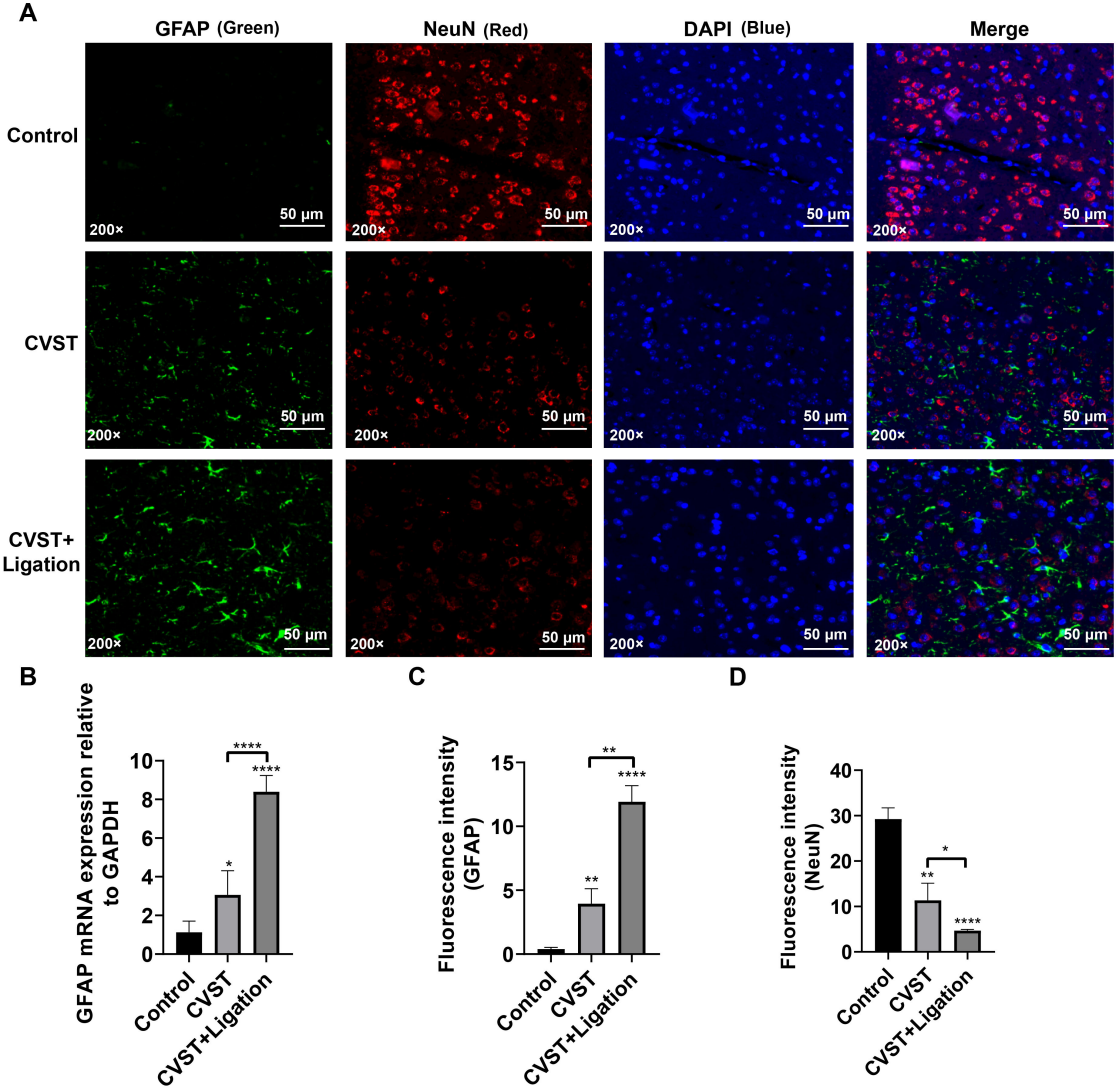
mLVs dysfunction exacerbates CVST-induced neuronal injury. **(A)** Immunofluorescence staining of GFAP and NeuN in mouse brain tissue (n=5). The CVST group exhibited reduced NeuN fluorescence intensity and increased GFAP-positive areas, indicating neuronal injury and astrocyte activation. In the CVST+Ligation group, NeuN signal loss was more pronounced and GFAP reactivity was further enhanced, demonstrating that lymphatic dysfunction exacerbated neuronal damage and intensified astrocyte activation. GFAP staining is shown in green, Neun staining is shown in red and DAPI staining is shown in blue. The scale bars represent 50 μm. **(B)** RT-qPCR analysis of GFAP expression in mouse brain tissue, confirming that lymphatic ligation enhanced CVST-induced astrocyte activation at the transcriptional level. **(C, D)** Quantification of GFAP and NeuN fluorescence intensity from **(A)** using ImageJ. Statistical analysis was performed using unpaired t-test in GraphPad Prism 8. *P < 0.05, **P < 0.01, ****P < 0.0001.

Analysis of GFAP and NeuN revealed upregulated GFAP and downregulated NeuN expression in both groups (**Figure 3**), indicating that both CVST and CVST+Ligation induce neuronal injury, with ligation further exacerbating CVST-induced damage. Additionally, immunofluorescence staining demonstrated elevated IBA1, iNOS (**Supplementary Figure 2**), and CD68 (**Supplementary Figure 3**) expression following CVST, with more pronounced upregulation in the ligation group, whereas CD206 expression remained unchanged (**Supplementary Figure 3**). Altered BAX and BCL2 immunofluorescence intensity (**Supplementary Figure 4**) further indicated enhanced apoptosis induction upon meningeal lymphatic dysfunction. These findings demonstrate that CVST triggers neuroinflammatory activation, leading to apoptosis and neuronal necrosis, which is further aggravated by meningeal lymphatic dysfunction induced by lymphatic ligation.

### 3.2 Using transcriptomic analysis, relevant alterations may be observed in endoplasmic reticulum stress/oxidative stress-related pathways following mLVs dysfunction.

**Figure 4:**
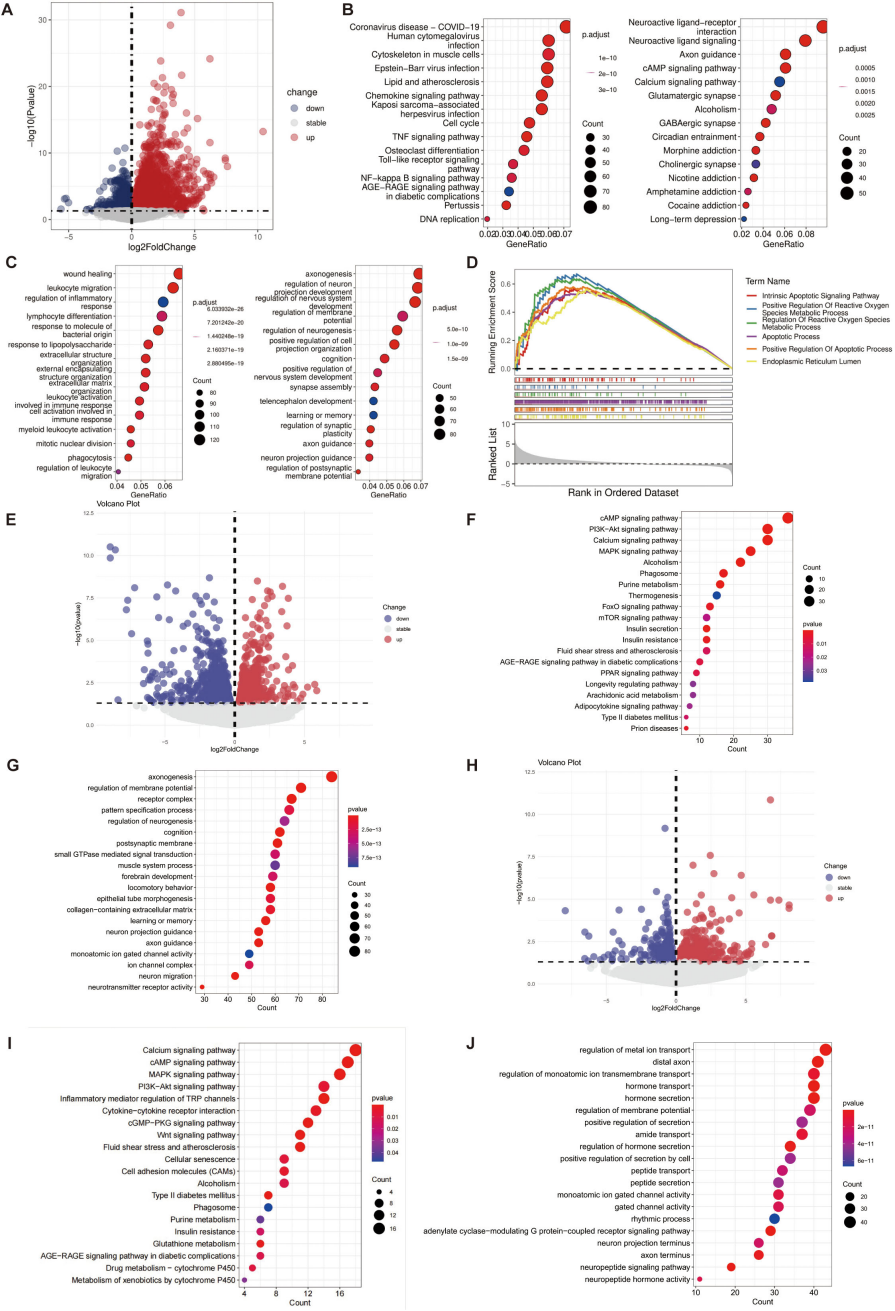
Relevant alterations may be observed in endoplasmic reticulum stress/oxidative stress-related pathways following mLVs dysfunction. **(A-D)** Pathway alterations between the CVST and sham operation groups were analyzed using the GSE245455 dataset. **(A)** DEGs between the CVST and Sham groups. **(B)** The enriched KEGG pathways for the up-regulated and down-regulated genes in the CVST versus Sham group. **(C)** The enriched GO pathways for the up-regulated and down-regulated genes in the CVST versus Sham group. **(D)** The GSEA analysis results of DEGs in the CVST group compared with the Sham group. **(E, F)** Pathway alterations after further analysis of meningeal lymphatic dysfunction were analyzed using our own data set. **(E)** The volcano plot shows DEGs results of the CVST+Ligation to the CVST. **(F)** The enriched KEGG pathways in the CVST+Ligation versus CVST. **(G)** The enriched GO pathways in the CVST+Ligation versus CVST. **(H)** The volcano plot shows DEGs results of the 4-PBA to the CVST+Ligation. **(I)** The enriched KEGG pathways in the 4-PBACVST+Ligation versus CVST+Ligation. **(J)** The enriched GO pathways in the 4-PBA versus CVST+Ligation.

To elucidate the mechanisms by which meningeal lymphatic dysfunction triggers neuroinflammatory activation, we performed transcriptomic analysis of dataset GSE245455. Compared to the sham surgery group, the CVST group exhibited a substantial number of differentially expressed genes (DEGs) (**Figure 4A**). KEGG enrichment analysis revealed that upregulated genes were significantly concentrated in inflammation and immunity-related pathways, such as cytokine-cytokine receptor interactions and viral protein-cytokine interactions (**Figure 4B**). Conversely, downregulated genes primarily involved neurofunctional pathways, including synaptic organization and neurotransmitter transmission (**Figure 4B**). GO enrichment results indicated that key biological process alterations induced by CVST included enhanced inflammatory responses and increased transcriptional regulatory activity (**Figure 4C**). GSEA findings further suggested that endoplasmic reticulum stress, oxidative stress, and inflammatory response pathways were significantly activated under CVST conditions (**Figure 4D**).

To investigate the role of meningeal lymphatics, we further utilized our transcriptomic data to compare gene expression profiles between CVST combined with lymphatic ligation and CVST alone models. KEGG pathway analysis revealed that the differential genes in the CVST ligation group were significantly enriched in stress and survival signaling pathways such as calcium signaling, PI3K-Akt, MAPK, and cAMP. Concurrently, FoxO signaling and AGE-RAGE signaling were specifically enriched, indicating the activation of antioxidant defense and oxidative stress responses (**Figure 4E, F**). GO analysis demonstrated that the differential genes were functionally concentrated in neural development and plasticity processes, including axonal occurrence, neurogenesis regulation, neuronal migration, and learning and memory. In terms of cellular localization, they were enriched in the postsynaptic membrane and ion channel complexes. Molecularly, these genes were associated with ion-gated channels and neurotransmitter receptor activity (**Figure 4G**). These results suggest that CVST ligation not only induced widespread cellular stress and metabolic dysregulation but also specifically affected the structural and functional integrity of neurons.

To validate the role of meningeal lymphatic vessels in endoplasmic reticulum stress/oxidative stress, we conducted further analysis after 4-PBA intervention. KEGG enrichment analysis revealed that the glutathione metabolism pathway was specifically enriched, while stress-related pathways such as calcium signaling, PI3K-Akt, and MAPK, as well as inflammation and cellular senescence-related pathways, remained significantly enriched (**Figure 4H, I**). GO results indicated that differentially expressed genes after 4-PBA treatment were primarily enriched in ion transport regulation, secretion processes, and neuronal synaptic terminal-related functions (**Figure 4J**). This suggests that 4-PBA may exert neuroprotective effects by modulating redox metabolism and neuronal secretion functions.

Collectively, these findings demonstrate that meningeal lymphatic dysfunction exacerbates transcriptional activation of endoplasmic reticulum stress/oxidative stress and associated apoptotic signaling pathways beyond CVST alone, revealing a potential mechanism for aggravating brain injury at the gene expression level.

### 3.3 Dysfunction of meningeal lymphatic vessels leads to elevated expression of autophagy-related pathways such as endoplasmic reticulum stress/oxidative stress

**Figure 5:**
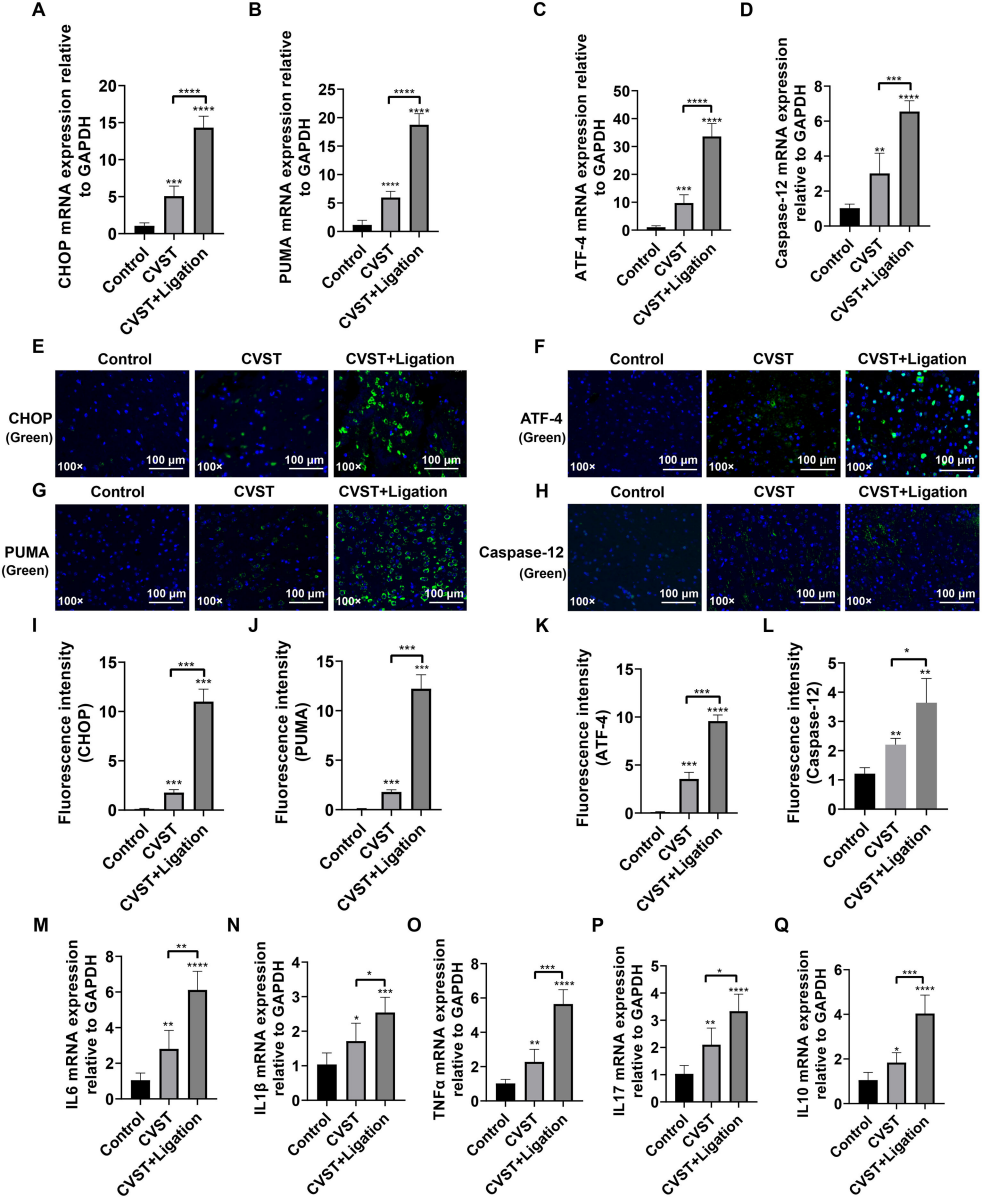
mLVs dysfunction exacerbates CVST-induced endoplasmic reticulum stress and inflammatory response. **(A–D)** RT-qPCR analysis of CHOP, PUMA, ATF4, and Caspase12 expression in mouse brain tissue. Expression levels were elevated in CVST mice and further increased in the CVST+ligation group. **(E–H)** Immunofluorescence staining of CHOP, PUMA, ATF4, and Caspase12 in mouse brain tissue (n=5). The staining patterns aligned with the gene expression trends, with the CVST+Ligation group displaying the strongest fluorescent signals. CHOP **(E)**, ATF4 **(F)**, PUMA **(G)**, and Caspase-12 **(H)** are stained green, and nuclei are stained with DAPI (blue). The scale bars represent 100 μm. **(I–L)** Quantification of fluorescence intensity from **(E-H)** using ImageJ. **(M–Q)** RT-qPCR analysis of inflammatory cytokines IL6, IL1β, TNFα, IL17, and IL10 in mouse brain tissue. These inflammatory markers were significantly upregulated in CVST, with further enhancement in the CVST+ligation group. Data were analyzed using unpaired t-test in GraphPad Prism 8.*P < 0.05, **P < 0.01, ***P < 0.001, ****P < 0.0001.

We validated the activation of the ER stress/oxidative stress pathway following meningeal lymphatic dysfunction in CVST mice using multiple approaches. RT-qPCR analysis revealed upregulated expression of CHOP, PUMA, ATF4, and Caspase-12 in both CVST and CVST+Ligation groups, suggesting induction of ER stress in brain tissue (**Figures 5A-D**). Immunofluorescence staining further corroborated the upregulation of these ER stress markers (**Figures 5E-L**). Additionally, RT-qPCR demonstrated elevated expression of IL-6, IL-1β, TNF-α, IL-17, and IL-10, indicating that CVST triggers neuroinflammation, which is exacerbated by ligation (**Figures 5M-Q**).

**Figure 6:**
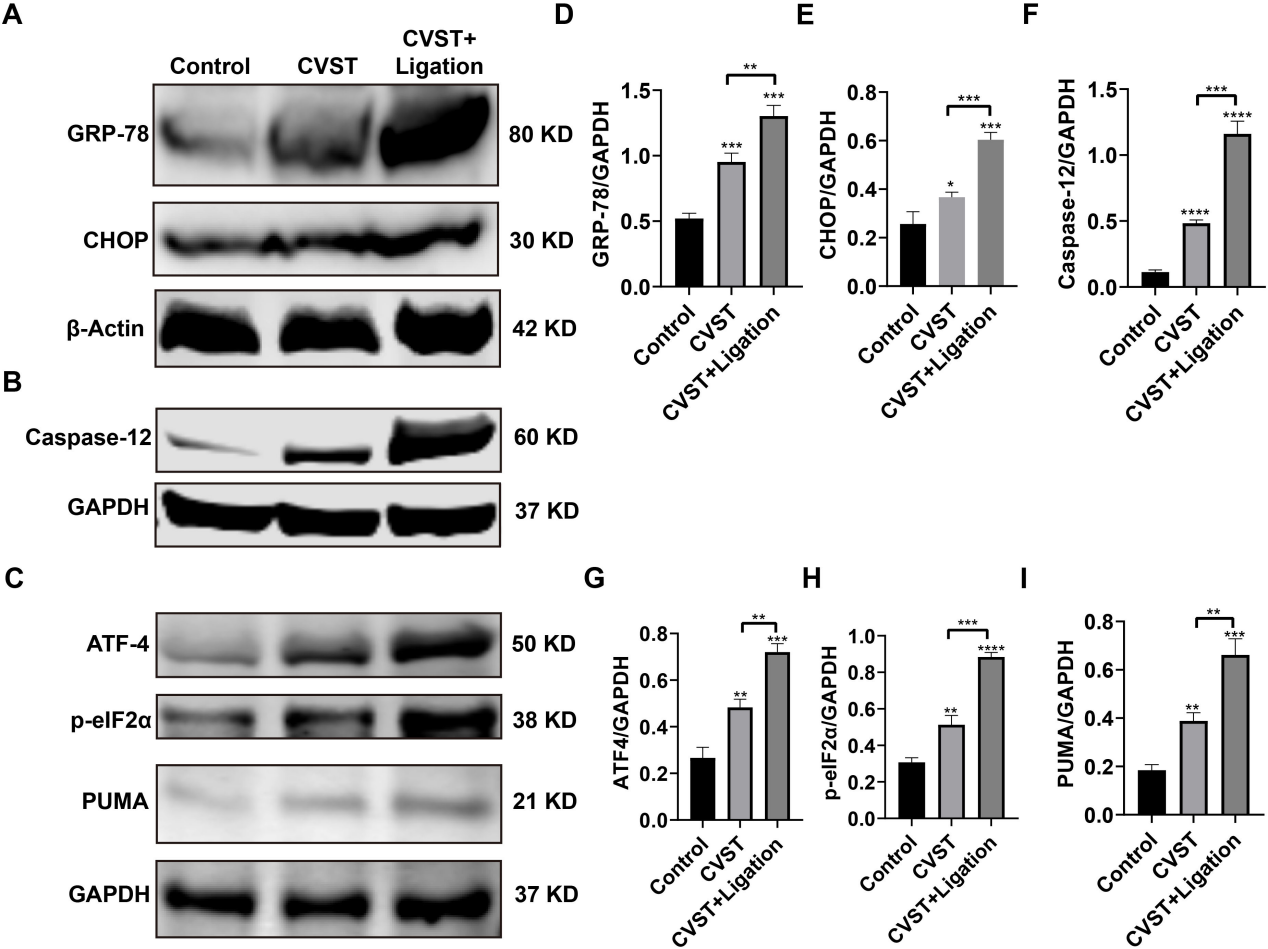
Lymphatic dysfunction exacerbates CVST-induced endoplasmic reticulum stress. **(A-C)** Western blot analysis of GRP78, CHOP, Caspase12, ATF4, p-eIF2α, and PUMA expression in mouse brain tissue post-induction (n=3). Expression of these proteins was upregulated in the CVST group compared with the control group and further significantly increased in the CVST+ligation group. **(D–I)** Quantification of band intensities from **(A-C)** was performed using ImageJ. Statistical analysis was conducted using unpaired t-test in GraphPad Prism 8.*P < 0.05, **P < 0.01, ***P < 0.001, ****P < 0.0001.

Compared with the Control group, both the CVST and CVST+Ligation groups exhibited significantly upregulated expression of GRP78, CHOP, Caspase-12, ATF4, p-eIF2α, and PUMA (**Figure 6**), indicating that both conditions induced ER stress. Notably, the CVST+Ligation group displayed even higher protein levels of these markers than the CVST group, demonstrating that ligation exacerbated CVST-induced ER stress. Additionally, immunofluorescence analysis of NLRP3 and IL-1β expression revealed significantly increased microglial pyroptosis in the ligation group (**Supplementary Figure 5**).

### 3.4 Suppression of endoplasmic reticulum stress leads to reduced expression of pathways associated with oxidative stress in CVST following meningolymphatic dysfunction

**Figure 7:**
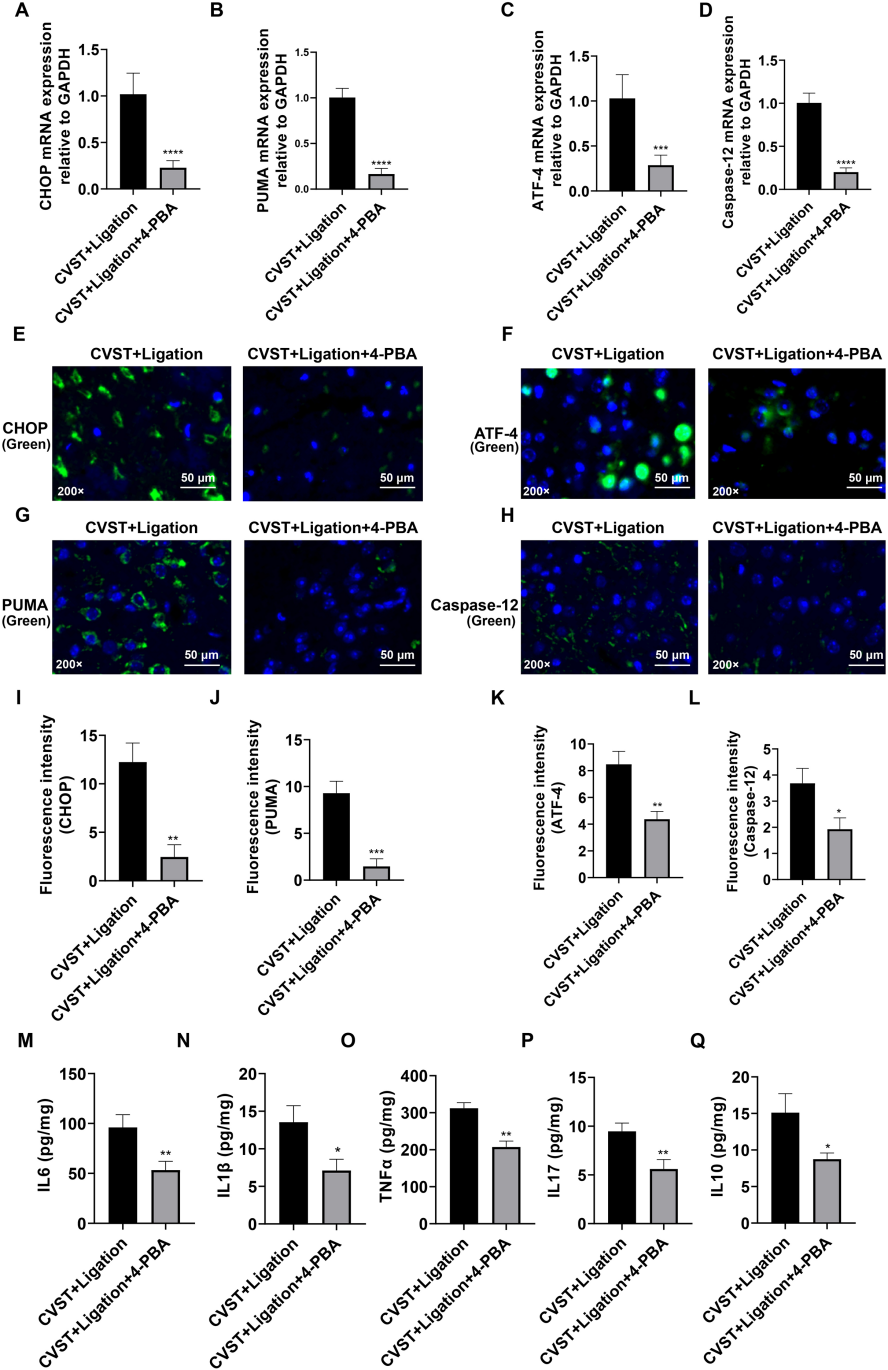
Inhibition of endoplasmic reticulum stress attenuates CVST+Ligation-induced inflammatory response. **(A-D)** RT-qPCR analysis of CHOP, PUMA, ATF4, and Caspase12 expression in mouse brain tissue. 4-PBA treatment significantly decreased the mRNA expression levels of pro-apoptotic factors CHOP **(A)**, PUMA **(B)**, ATF4 **(C)**, and Caspase12 **(D)** compared with the CVST+Ligation model group. **(E-H)** Immunofluorescence staining of CHOP, PUMA, ATF4, and Caspase12 in mouse brain tissue (n=5). Fluorescent signals (green) for CHOP **(E)**, PUMA **(F)**, ATF4 **(G)**, and Caspase12 **(H)** were markedly increased in the CVST+Ligation group, while 4-PBA treatment reduced their expression. The scale bars represent 50 μm. **(I-L)** Quantification of fluorescence intensity from **(E-H)** using ImageJ. **(M-Q)** ELISA analysis of IL6, IL1β, TNFα, IL17, and IL10 levels in mouse brain tissue. Pro-inflammatory cytokine levels were significantly elevated in the CVST+Ligation group and were markedly reduced by 4-PBA intervention. Statistical analysis was conducted using unpaired t-test in GraphPad Prism 8. *P < 0.05, **P < 0.01, ***P < 0.001, ****P < 0.0001.

**Figure 8:**
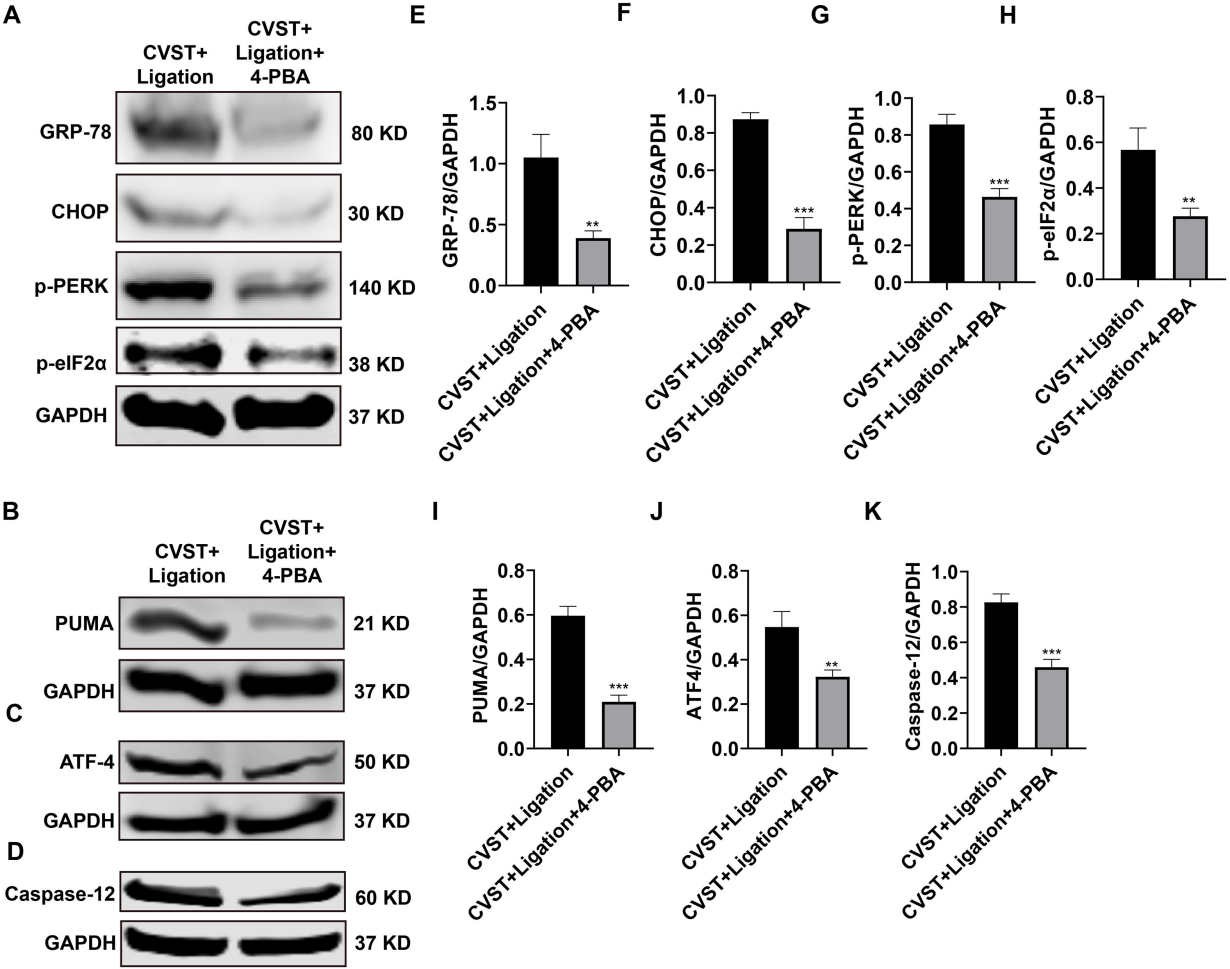
4-PBA treatment effectively inhibits CVST+Ligation-induced endoplasmic reticulum stress in mouse brain tissue. **(A-D)** Western blot analysis of GRP78, CHOP, p-PERK, p-eIF2α, PUMA, ATF4, and Caspase12 expression in mouse brain tissue following 4-PBA intervention (n=3), showing downregulation of these endoplasmic reticulum stress markers. **(E-K)** Quantification of band intensities using ImageJ and statistical analysis by unpaired t-test in GraphPad Prism 8 demonstrated that 4-PBA intervention significantly downregulated the expression of all these endoplasmic reticulum stress-related proteins compared with the CVST+Ligation model group. **P < 0.01, ***P < 0.001.

**Figure 9:**
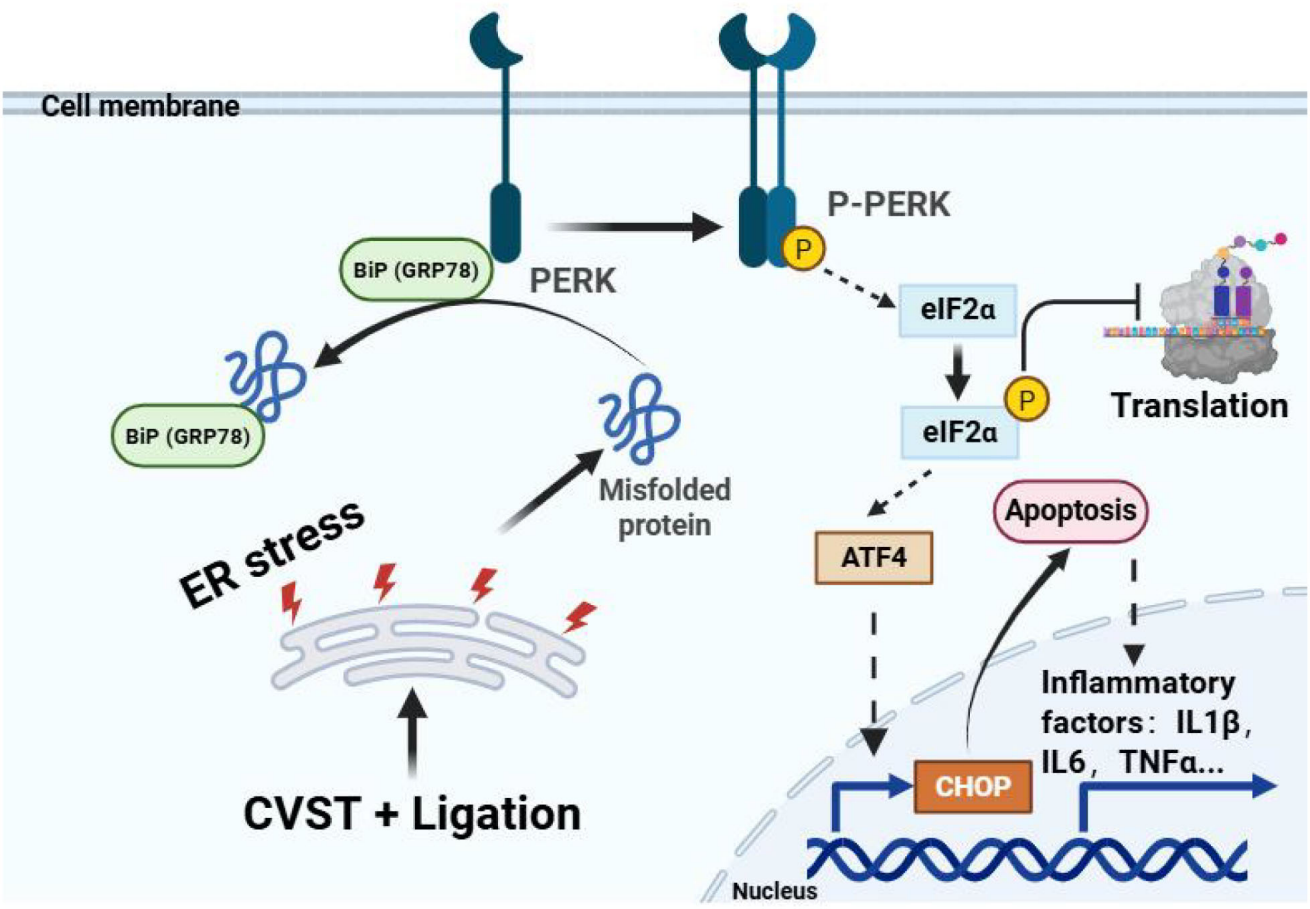
The schematic diagram demonstrates that dysfunction of meningeal lymphatics can exacerbate neuroinflammation through the endoplasmic reticulum stress/oxidative stress pathway, thereby worsening brain injury following CVST.

To further validate activation of the ER stress/oxidative stress pathway following lymphatic dysfunction, we employed 4-PBA as an ER stress inhibitor. RT-qPCR and immunofluorescence analysis demonstrated that 4-PBA treatment significantly attenuated the expression of CHOP, ATF4, PUMA, and Caspase-12 (**Figures 7A-L**). Consistently, ELISA revealed that 4-PBA markedly reduced the levels of IL-6, IL-1β, TNF-α, IL-17, and IL-10 (**Figures 7M-Q**). These findings indicate that inhibition of ER stress by 4-PBA effectively mitigates neuroinflammation induced by CVST+Ligation. Furthermore, they suggest that ligation exacerbates CVST-induced brain injury by aggravating ER stress. Western blotting confirmed that 4-PBA treatment significantly decreased the protein levels of GRP78, CHOP, p-PERK, p-eIF2α, PUMA, ATF4, and Caspase-12 (**Figure 8**), demonstrating that 4-PBA effectively suppresses ER stress in brain tissue triggered by CVST+Ligation (**Figure 9**). 

## 4 Discussion

The discovery of mLVs transformed the traditional understanding of neuroimmunology. These vessels are essential for clearing metabolic waste, circulating cerebrospinal fluid, and maintaining central nervous system homeostasis. A growing body of evidence suggests that mLVs play a significant role in the onset and progression of various neurological disorders. In the present study, we observed marked exacerbation of brain injury in CVST mice with mLV dysfunction. Concurrently, markers of ER stress and oxidative stress were significantly upregulated following mLV dysfunction. Pharmacological inhibition of ER stress alleviated brain injury in CVST mice with impaired mLVs. Collectively, these findings indicate that mLV dysfunction aggravates CVST-induced brain injury via a combined mechanism involving ER and oxidative stress pathways.

Dysfunction of the meningeal lymphatic system can influence the development and progression of neurological disorders. This system plays a key role in neurodegenerative diseases such as Alzheimer’s and Parkinson’s disease. Impaired meningeal lymphatic drainage hinders the removal of amyloid-β, promoting neuroinflammation and progressive neuronal loss, thereby exacerbating Alzheimer’s disease pathology. Treatment with vascular endothelial growth factor C (VEGF-C) has been shown to alleviate these symptoms (24, 25). Furthermore, a clinical trial investigating deep cervical lymphatic-venous anastomosis for Alzheimer’s disease was based on the theory of restoring meningeal lymphatic function (26). Zou et al. (27) demonstrated that ligation of the deep cervical lymph nodes in A53T mice exacerbates α-synuclein aggregation in the brain. This pathological process was accompanied by glial proliferation, increased pro-inflammatory cytokine expression, and elevated tau protein levels. Meningeal lymphatic function also affects outcomes in immune-mediated and infectious diseases of the nervous system. Louveau et al. (28) demonstrated that improving meningeal lymphatic function alleviates the clinical symptoms of experimental autoimmune encephalomyelitis in affected mice. Li et al. (29) observed that mLVs promote the clearance of intracranial viruses and play a crucial role in the pathophysiological responses to cerebrovascular disease and traumatic brain injury. Wang et al. (30) further discovered that, in rats with intraventricular hemorrhage, mLVs remove ferritin from the ventricles, thereby reducing sterile inflammation and mitigating the development of hydrocephalus. Yanev et al. (12) observed that hypoplasia of the mLVs exacerbated the severity of post-stroke brain injury in an arterial stroke mouse model. Similarly, Bolte et al. (11) demonstrated that impaired meningeal lymphatic function worsens traumatic brain injury. In our study, we ligated the deep cervical lymph nodes in CVST mice to further aggravate meningeal lymphatic dysfunction. Compared with unligated CVST mice, those subjected to lymph node ligation exhibited more pronounced behavioral deficits and more extensive brain injury, along with significantly increased cerebral cell apoptosis and neuronal damage. Consistent with observations in other neurological disorders, our findings suggest that impaired meningeal lymphatic function amplifies the extent of brain injury after CVST. The meningeal lymphatic system plays a key role in regulating neuroinflammatory responses within the brain by draining the cerebrospinal fluid and clearing metabolic waste products under both physiological and pathological states. This process profoundly influences the pathophysiological processes underlying neurological disorders. The mLV network facilitates the entry of central immune cells such as dendritic cells (DCs), B lymphocytes, T cells, and neutrophils into the peripheral immune system. This process induces self-reactive T-cell responses and regulates peripheral immunity (9, 31). Louveau et al. (28) demonstrated the presence of DCs within the mLV system. Following the injection of labelled DCs into the cisterna magna, they observed that these cells drained via the mLV network and participated in the central immune response. B lymphocytes in the meninges can migrate to the cervical lymph nodes and participate in intracranial immune responses (32). Microglia highly express the chemokine CCL21, which binds to the CCR7 receptor on the surface of immune cells, mediating the migration of T cells from the meninges to the deep cervical lymph nodes (28). Rustenhoven et al. (33) reported that single-cell sequencing of meningeal lymphatic endothelial monocytes in aged mice revealed an increase in interferon-γ (IFN-γ) within the aged meninges due to T-cell accumulation, which led to brain injury. Concurrently, inflammatory stimuli such as TNF-α and IL-1β can activate the NF-κB signaling pathway in meningeal lymphatic endothelial cells, inducing the expression of adhesion molecules, including Intercellular Adhesion Molecule-1 and Vascular Cell Adhesion Molecule-1, and thereby promoting immune cell recruitment and transmembrane migration (34). Our results suggest that following meningeal lymphatic dysfunction, CVST mice exhibit significantly increased cerebral cell apoptosis, alongside markedly elevated expression of IBA1 and CD68. At this stage, CVST mice demonstrate heightened pro-inflammatory levels, indicating that the meningeal lymphatic system influences brain injury in CVST mice by modulating inflammatory responses.

CVST obstructs cerebral venous outflow, elevates intracranial pressure, and impairs the drainage of extracellular fluid. The resulting accumulation of fluid within the brain parenchyma leads to cerebral edema. Concurrently, disruption of the blood-brain barrier triggers an inflammatory response. These three fundamental pathological processes form a vicious cycle that amplifies brain tissue damage. Inflammatory and immune mechanisms play crucial roles in the pathogenesis of CVST and strongly influence its clinical prognosis (35, 36). Jin et al. (2) observed that neutrophil extracellular traps participate in autophagy and promote thrombogenesis in patients with CVST. Inflammatory cells such as microglia, astrocytes, and neutrophils contribute to the pathophysiology of brain injury following CVST (36). Ding et al. (20) demonstrated that inhibition of the cGAS-STING axis reduces inflammasome activation and microglial pyroptosis in CVST mice, thereby mitigating brain injury. In another study, Ding et al. (23) used RNA sequencing to analyze transcriptional alterations in the cerebral cortex following CVST and observed a marked increase in inflammatory response pathways. Through analysis of these transcriptomic data, we identified that pathways related to ER stress, oxidative stress, and inflammation were significantly activated in the CVST state. Activation of the ER and oxidative stress pathways constitutes a key neuroinflammatory mechanism underlying CVST-induced brain injury (16).

To investigate alterations in endoplasmic reticulum (ER) and oxidative stress pathways in CVST mice with meningeal lymphatic dysfunction, we analyzed their transcriptomic levels. KEGG pathway enrichment analysis revealed that the AGE-RAGE signaling pathway, which is associated with oxidative stress, was persistently activated throughout the disease process. Concurrently, the core antioxidant defense pathway (FoxO signaling) was induced specifically in the CVST and CVST+Ligation groups. However, after treatment with the ER stress inhibitor 4-PBA, enrichment of this pathway disappeared and was replaced by significant activation of the glutathione metabolism pathway. Additionally, the calcium signaling pathway, which serves as a critical bridge between ER stress and oxidative stress, was highly enriched in both comparison groups. GO enrichment analysis revealed that differentially expressed genes were significantly enriched in pathways related to axonal structure, ion channel function, neuropeptide secretion, and cognition. These pathways are closely associated with maintaining neuronal homeostasis, and their dysregulation is a classic downstream event of ER and oxidative stress. Furthermore, the expression profile of secretion-related pathways exhibited a significant reversal following treatment with the ER stress inhibitor 4-PBA, suggesting that ER stress plays a pivotal role in neuronal damage in this model. Taken together, these data demonstrate that CVST combined with ligation treatment induces significant ER stress, leading to disruption of calcium homeostasis and driving oxidative stress. Conversely, 4-PBA alleviates ER stress, thereby restoring the cell’s redox balance. Therefore, meningeal lymphatic dysfunction may involve inflammatory and cytokine-mediated signaling regulation and be associated with endoplasmic reticulum stress and apoptosis processes.

Oxidative stress activates inflammasomes, particularly NLRP3, through excessive generation of reactive oxygen species. Inflammasomes, including NLRP3, act as multiprotein receptors that promote the maturation of IL-1β and IL-18 and induce pyroptosis, thereby participating in inflammatory responses and immune regulation. ER stress denotes the cellular response to misfolded or unfolded proteins within the ER that exceed their folding capacity. Oxidative stress and ER stress interact closely, each capable of provoking the other. Our transcriptomic analysis indicates that meningeal lymphatic dysfunction likely disrupts this equilibrium, thereby perpetuating and amplifying a vicious cycle that culminates in more severe neuronal apoptosis and neuroinflammation. Therefore, using molecular biology techniques including western blotting, polymerase chain reaction, and immunofluorescence, we examined the pathways associated with ER stress and oxidative stress in both the CVST and lymphatic ligation groups. We observed a marked elevation of ER and oxidative stress markers following lymphatic dysfunction. Subsequent inhibition of ER stress with 4-phenylbutyric acid effectively reversed these abnormal molecular changes. These results demonstrate that upregulation of ER and oxidative stress pathways following meningeal lymphatic dysfunction exacerbates brain injury in CVST mice and further supports our central hypothesis. Endoplasmic reticulum (ER) stress was primarily driven by the PERK-eIF2α-ATF4-CHOP signaling pathway (37). During ER stress-mediated apoptosis, key markers such as CHOP, p-IRE1α, p-PERK, and caspase-12 were significantly upregulated at early stages, subsequently inducing or modifying TXNIP expression, a change closely associated with alterations in neurological function and brain injury (16). In contrast, although phosphorylation/cleavage of IRE1α or ATF6 also increased, their peak expression occurred later (≥48 h), and specific inhibition of these branches did not significantly affect acute-phase infarct volume, suggesting their greater involvement in delayed repair or chronic inflammation (38). Therefore, this study focused on detecting key indicators of the PERK-eIF2α-ATF4-CHOP pathway. Results showed significant elevation of these indicators on the second day post-modeling, consistent with previous findings. In CVST mice with meningeal lymphatic dysfunction, the upregulation of these pathway markers was even more pronounced, indicating that such dysfunction may exacerbate brain injury by modulating this pathway. Furthermore, inhibition of ER stress using 4-PBA effectively reversed the aberrant changes in these molecular markers. These results suggest that meningeal lymphatic dysfunction enhances ER stress/oxidative stress pathway activation, thereby aggravating brain injury in CVST mice.

Our study has certain limitations. First, ligation of the deep cervical lymph nodes to simulate meningeal lymphatic dysfunction does not fully replicate the complex alterations in lymphatic drainage observed in human cases of CVST; moreover, the ligation procedure itself may induce local inflammation and thus introduce potential confounding effects. Second, we focused primarily on the core ER- and oxidative stress pathways after CVST-associated lymphatic dysfunction and did not fully explore the precise interactions with other key pathways, such as autophagy and additional cell-death programs. Additionally, it should be noted that this study employed only male mice. Future research should explore the impact of gender differences on mLV function and CVST prognosis.

In future studies, we may consider employing conditional gene knockout mice (such as those with specific deletion of key genes in lymphatic endothelial cells) or VEGF-C-mediated lymphatic-specific hyperplasia models to more precisely regulate meningeal lymphatic function. These approaches will enable validation of the implicated mechanisms and elucidation of upstream and downstream molecular connections among meningeal lymphatics, oxidative stress, NLRP3 inflammasome activation, and pyroptosis. Ultimately, these investigations will strengthen the mechanistic rationale for novel therapeutic approaches targeting meningeal lymphatic function in CVST.

## 5 Conclusions

Dysfunction of the meningeal lymphatic system represents a key contributing factor to exacerbated brain injury after CVST. Our experimental findings demonstrate that impaired lymphatic drainage significantly worsens neurological deficits and amplifies brain tissue pathology, inflammatory responses, and cellular apoptosis. Furthermore, we identified ER and oxidative stress pathways as pivotal mediators of this heightened brain injury. These findings provide crucial theoretical and experimental support for novel therapeutic approaches that aim to protect or enhance meningeal lymphatic function as an approach to mitigate CVST-related brain injury.

## Data Availability

The data presented in this study have been deposited in the GEO repository, with the accession number GSE317334.
