# Supplementary material for: Meningeal lymphatic vessel dysfunction exacerbates brain injury in CVST mice via endoplasmic reticulum and oxidative stress pathways
Source: Front Immunol. 2026 Jan 30;17:1745066. doi: 10.3389/fimmu.2026.1745066 (PMC12900663; doi:10.3389/fimmu.2026.1745066)
Supplement: Supplementary file 1 [file Supplementaryfile1.doc]

**Supplementray**

**
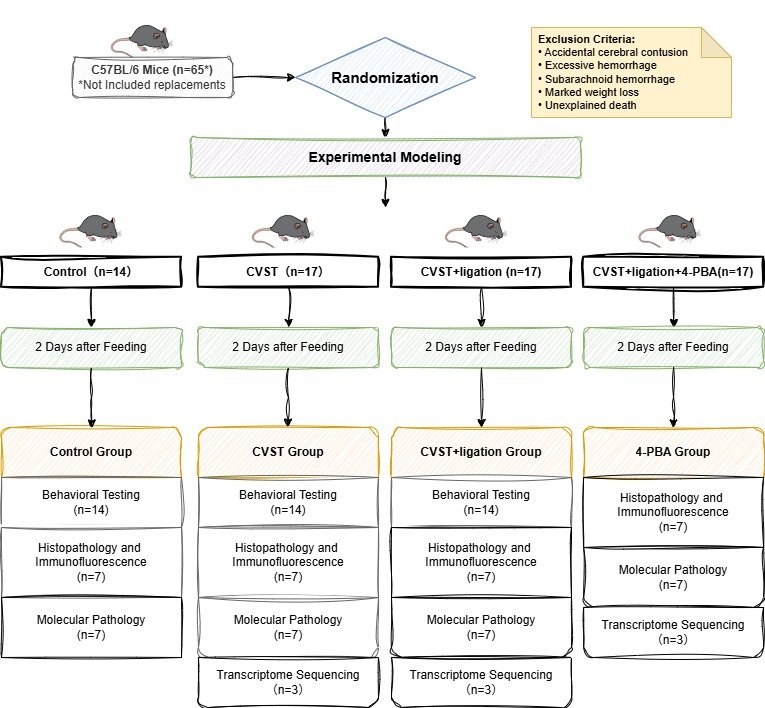
**

**Supplementary Fig.1 Flowchart and Allocation of Experimental Animals ：The sample quantity in the figure represents the number of mice.**


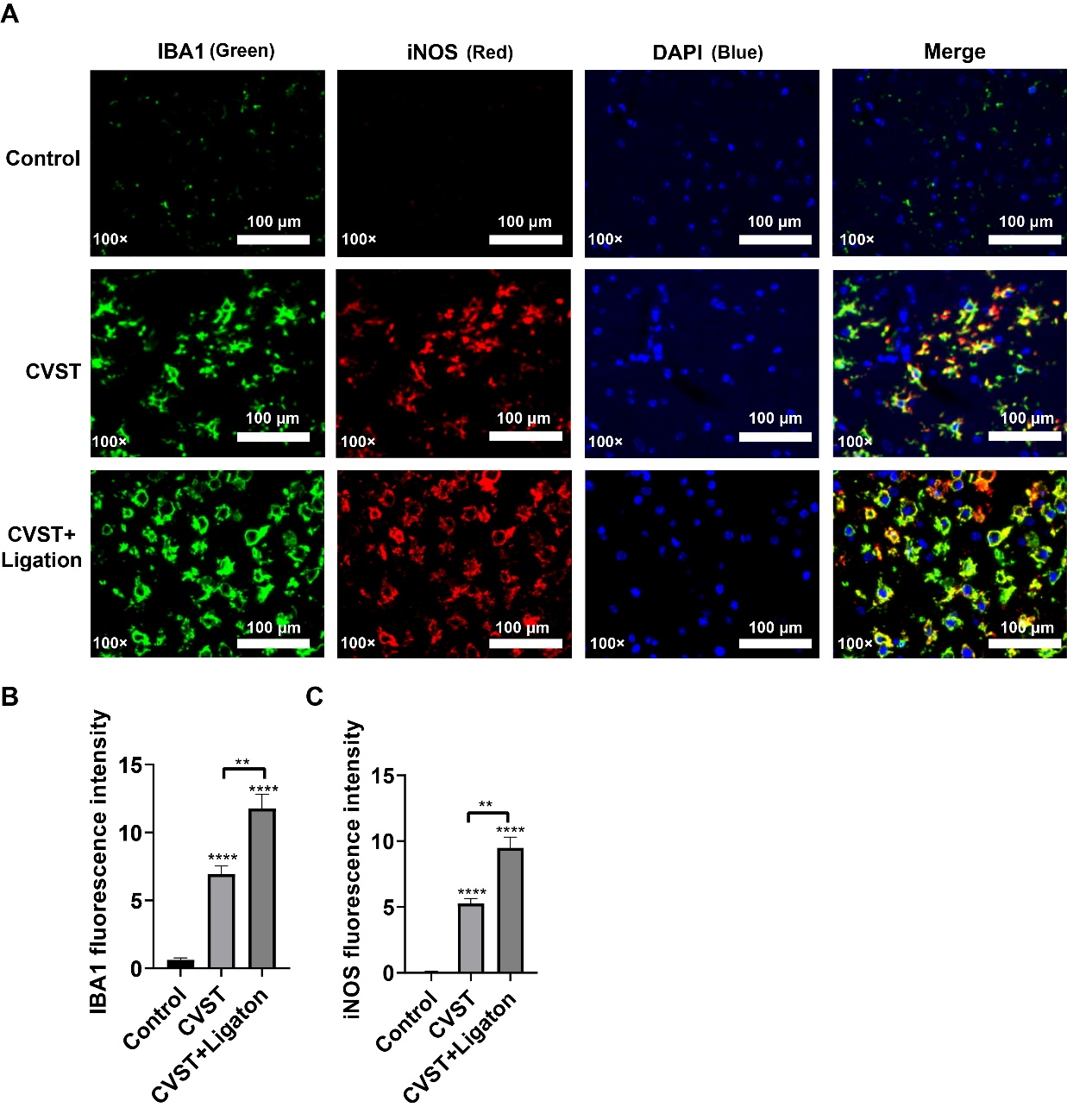


**Supplementary Fig.2 mLVS dysfunction exacerbates neuroinflammatory responses in CVST.**

A: Immunofluorescence staining of IBA1 and iNOS co-expression in mouse brain tissue (n=5). Increased fluorescence intensity of IBA1 and iNOS was observed in the CVST group, indicating activation of post-CVST neuroinflammation, which was further enhanced in the CVST+Ligation group, suggesting that lymphatic dysfunction exacerbated neuroinflammatory responses. IBA1 is shown in green, iNOS in red, and nuclei are counterstained with DAPI (blue). The scale bars represent 100 μm.

B-C: Quantification of IBA1 and iNOS fluorescence intensity from (A) using ImageJ. Statistical analysis was performed using unpaired t-test in GraphPad Prism 8. ***P*<0.01，*****P*<0.0001.


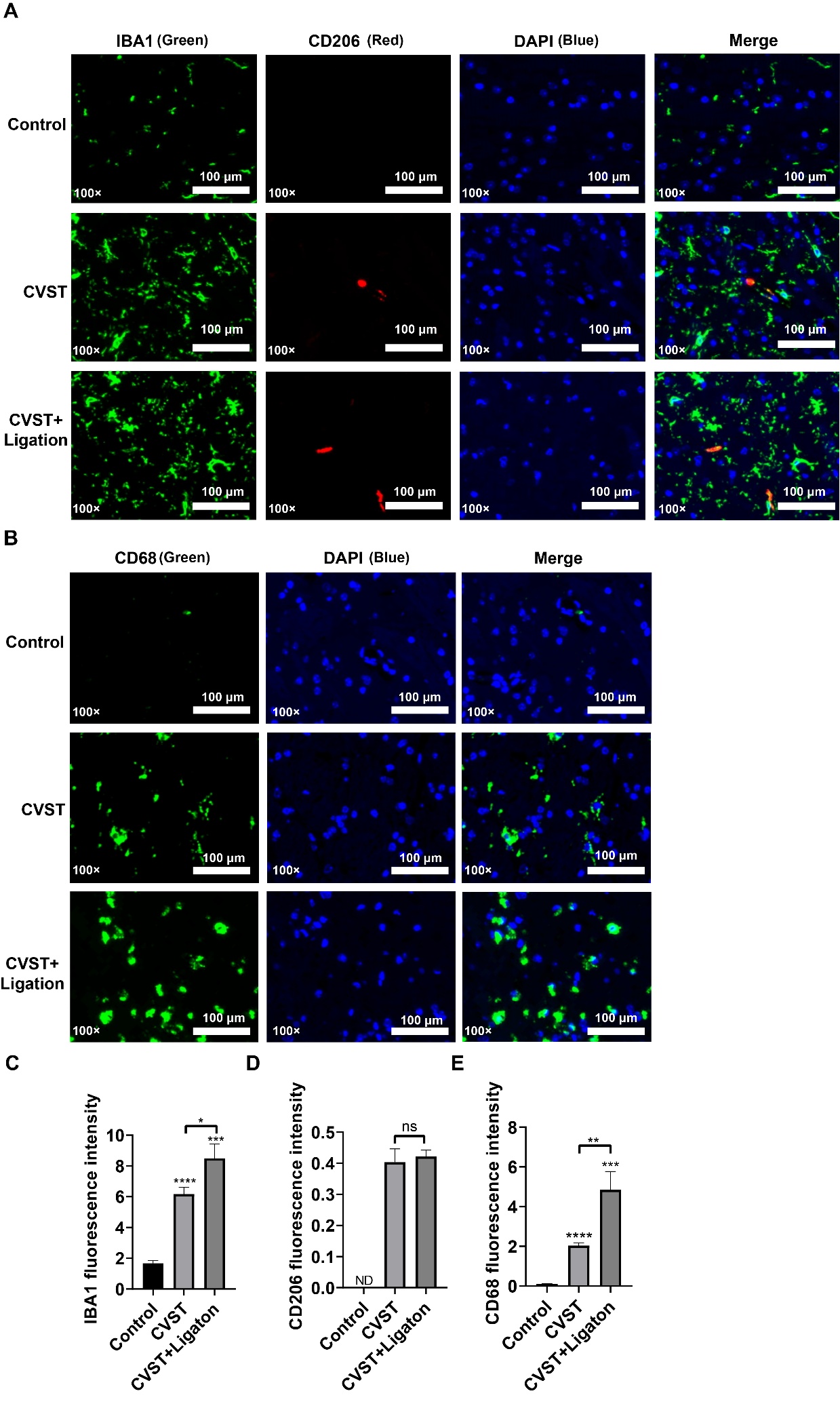


**Supplementary Fig.3 mLVs dysfunction exacerbates neuroinflammatory responses in CVST.**

A: Immunofluorescence staining of IBA1 and CD206 co-expression in mouse brain tissue (n=5). Increased fluorescence intensity of IBA1 and CD206 was observed in the CVST group, indicating activation of post-CVST neuroinflammation, which was further enhanced in the CVST+Ligation group, suggesting that lymphatic dysfunction exacerbated neuroinflammatory responses. IBA1 is shown in green, CD206 in red, and nuclei are counterstained with DAPI (blue). The scale bars represent 100 μm.

B: Immunofluorescence staining of CD68 in mouse brain tissue (n=5). Fluorescence intensity was increased in the CVST group, indicating neuroinflammatory activation after CVST, and was further enhanced in the CVST + Ligation group, suggesting that lymphatic dysfunction exacerbates neuroinflammation. CD68 is shown in green; nuclei are counterstained with DAPI (blue). Scale bars: 100 μm.

C-E: Quantification of IBA1 CD26 and CD68 fluorescence intensity from (A-B) using ImageJ. Statistical analysis was performed using unpaired t-test in GraphPad Prism 8. **P*<0.05，***P*<0.01，****P*<0.001 and *****P*<0.0001. ND: Not detected. ns: not significant
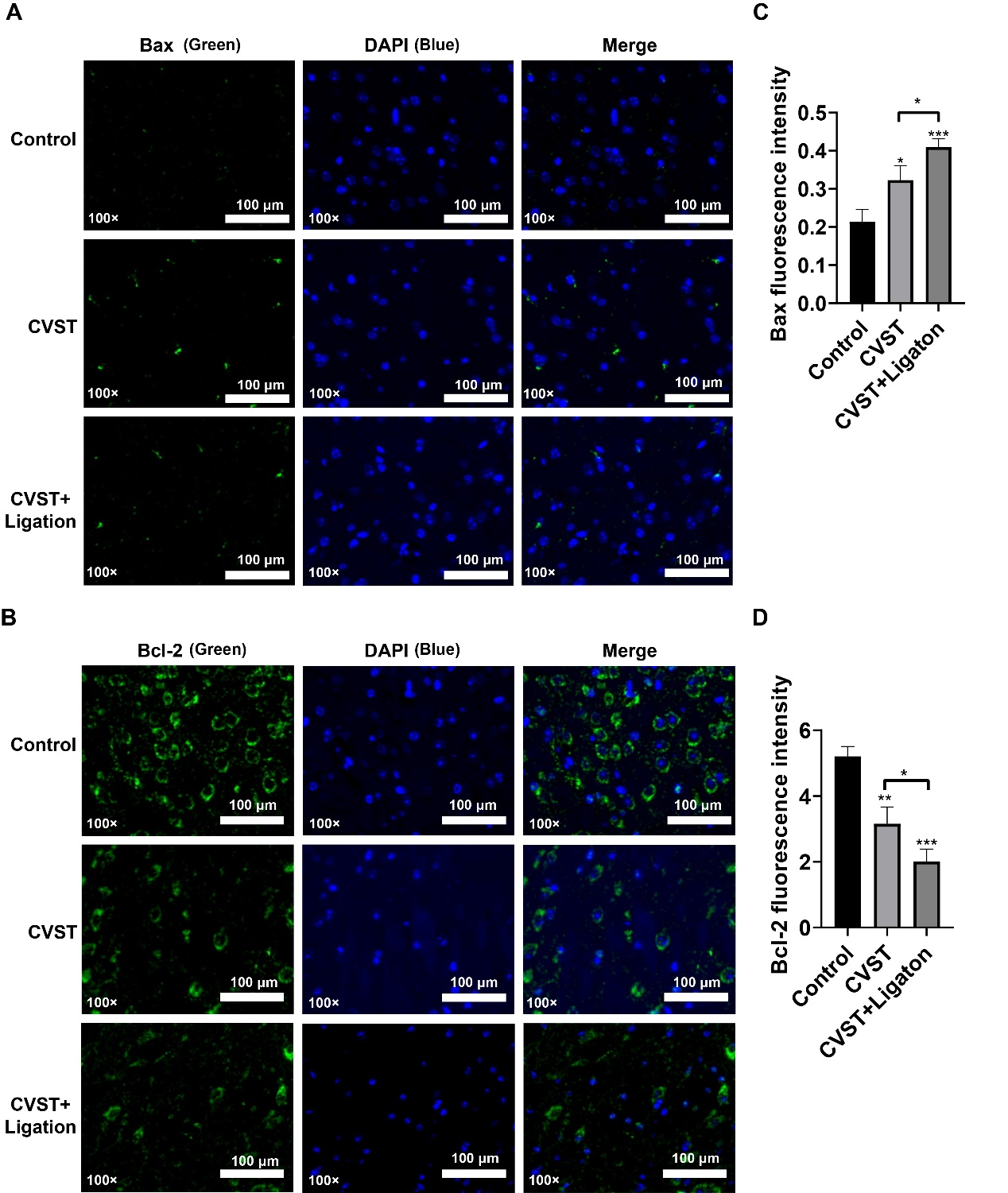


**Supplementary Fig.4 mLVs dysfunction exacerbates CVST-induced apoptosis.**

A: Immunofluorescence staining of Bax expression in mouse brain tissue (n=5). Bax fluorescence intensity was increased in both groups, with a more pronounced elevation in the CVST+Ligation group. Bax is shown in green; nuclei are counterstained with DAPI (blue). Scale bars: 100 μm.

B: Immunofluorescence staining of Bcl-2 expression in mouse brain tissue (n=5). Bcl-2 fluorescence intensity was decreased in both groups, with a more significant reduction in the CVST+Ligation group. These findings further demonstrate that CVST promotes apoptosis, which is exacerbated by lymphatic dysfunction in CVST mice. Bcl-2 is shown in green; nuclei are counterstained with DAPI (blue). Scale bars: 100 μm.

C-D: Quantification of Bax and Bcl-2 fluorescence intensities from (A) and (B) using ImageJ. Statistical analysis was performed using unpaired t-test in GraphPad Prism 8. **P*<0.05，***P*<0.01 and ****P*<0.001。


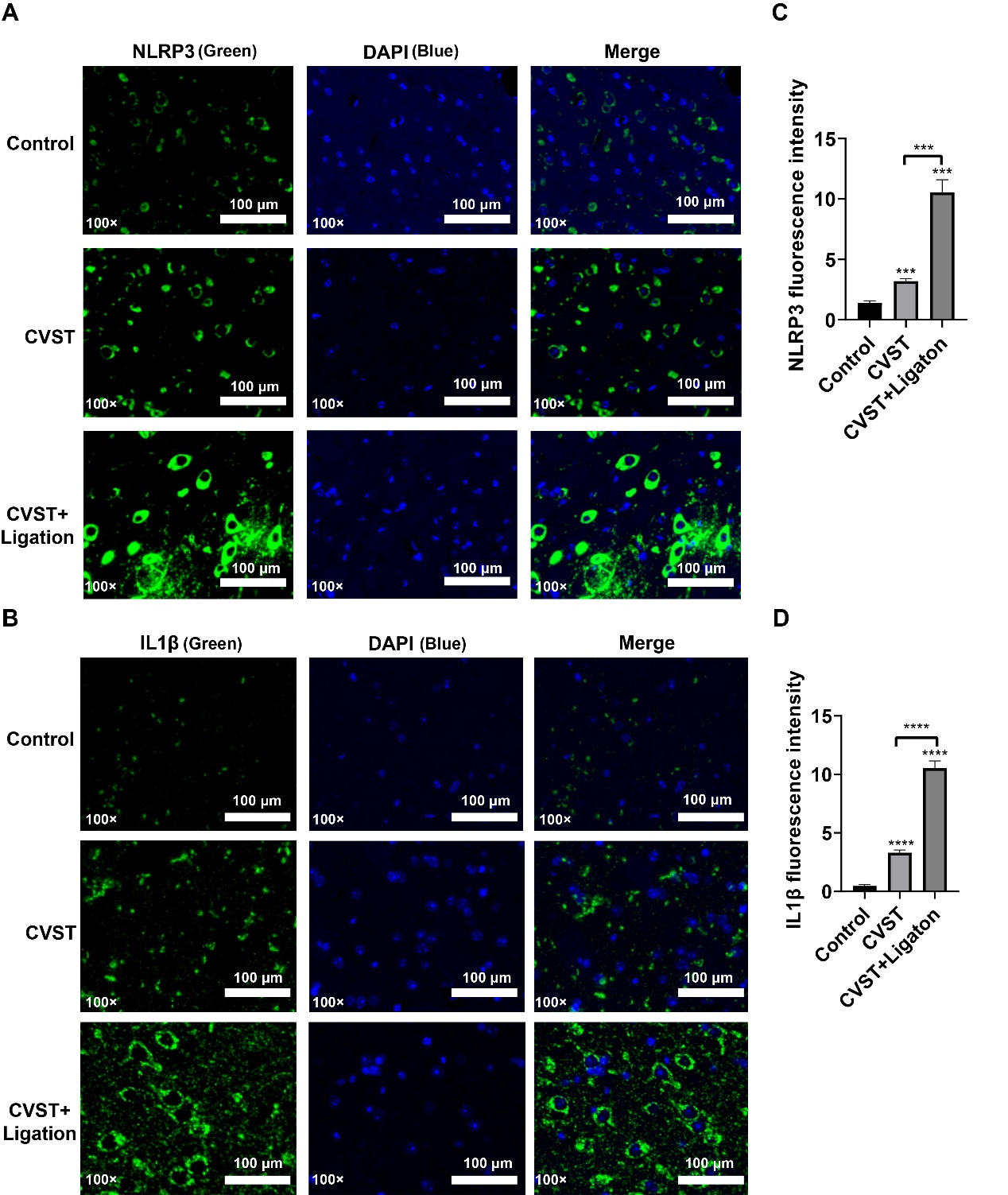


**Supplementary Fig.5 mLVs dysfunction exacerbates CVST-induced pyroptosis.**

A: Immunofluorescence staining of NLRP3 in mouse brain tissue (n=5). NLRP3 fluorescence intensity was increased in both groups, with a more pronounced elevation in the CVST+Ligation group. NLRP3 is shown in green; nuclei are counterstained with DAPI (blue). Scale bars: 100 μm.

B: Immunofluorescence staining of IL-1β in mouse brain tissue (n=5). IL-1β fluorescence intensity was enhanced in both groups, with a more significant increase in the CVST+Ligation group. These findings further demonstrate that CVST promotes microglial pyroptosis, which is markedly exacerbated by lymphatic dysfunction. NLRP3 is shown in green; nuclei are counterstained with DAPI (blue). Scale bars: 100 μm.

1. D: Quantification of NLRP3 and IL-1β fluorescence intensities using ImageJ. Statistical analysis was performed using unpaired t-test in GraphPad Prism 8. ****P*<0.001 and *****P*<0.0001。

| **Primer** | **Sequence** |
| --- | --- |
| **GFAP-Forward** | **5' TGGAGAGAAAGGTTGAATCGCT 3'** |
| **GFAP-Reverse** | **5' ACCGATACCACTCCTCTGTCTCTT 3'** |
| **CHOP-Forward** | **5'CACATCCCAAAGCCCTCG3'** |
| **CHOP-Reverse** | **5'TGGGCACTGACCACTCTGTTT3'** |
| **PUMA-Forward** | **5'CACCAGCCCAGCAGCACTT3'** |
| **PUMA-Reverse** | **5'TGAGGTCGTCCGCCATCC3'** |
| **ATF4-Forward** | **5'GGCCATCTCCCAGAAAGTTTAAT3'** |
| **ATF4-Reverse** | **5'AATGTAAGCAGCAGAGTCAGGC3'** |
| **Caspase12-Forward** | **5'AGCACCAGTCCTCAGACAGCAC3'** |
| **Caspase12-Reverse** | **5'GGGTTTGTTTCTCAGACTCCGA3'** |
| **IL1β-Forward** | **5'GAAGGGCTGCTTCCAAACCT3'** |
| **IL1β-Reverse** | **5'GCTTCTCCACAGCCACAATG3'** |
| **TNFα-Forward** | **5'CTCTTCTCATTCCTGCTTGTGG3'** |
| **TNFα-Reverse** | **5'CTCCACTTGGTGGTTTGTGAGT3'** |
| **IL17-Forward** | **5'CTCAGACTACCTCAACCGTTCC3'** |
| **IL17-Reverse** | **5'CTTTCCCTCCGCATTGACAC3'** |
| **IL10-Forward** | **5'GACAACATACTGCTAACCGACTC3'** |
| **IL10-Reverse** | **5'GCCGCATCCTGAGGGTCT3'** |
| **IL6-Forward** | **5'CTTCCATCCAGTTGCCTTCTTG3'** |
| **IL6-Reverse** | **5'CTCATTTCCACGATTTCCCAGA3'** |

**Supplementary Tab.1 The *PCR primer sequences***
